# Phenotypic Stability of *Zea mays* Grain Yield and Its Attributing Traits under Drought Stress

**DOI:** 10.3389/fpls.2017.01397

**Published:** 2017-08-22

**Authors:** Fawad Ali, Muhammad Ahsan, Qurban Ali, Naila Kanwal

**Affiliations:** ^1^Department of Plant Breeding and Genetics, University of Agriculture Faisalabad Faisalabad, Pakistan; ^2^Centre of Excellence in Molecular Biology, University of the Punjab Lahore, Pakistan

**Keywords:** maize hybrids, phenotypic stability, phenotypic and genotypic correlations, path coefficient analysis, bi-plot

## Abstract

Phenotypic stability under stress environment facilitate the fitness of genotype and opens new horizons to explore the cryptic genetic variation. Variation in tolerance to drought stress, a major grain yield constraint to global maize production, was identified, at the phenotypic and genotypic level. Here we found a prominent hybrid H_9_ that showed fitness over four growing seasons for grain yield under water stress conditions. Genotypic and phenotypic correlation of yield attributing traits over four seasons demonstrated that cobs per plant, 100 seed weight, number of grains rows per cob, total dry matter, cob diameter had positive association (*r*^2^ = 0.3–0.9) to grain yield. The perturbation was found for chlorophyll content as it showed moderate to strong association (*P* < 0.01) over four seasons, might be due to environment or genotype dependent. Highest heritability (95%) and genetic advance (79%) for grain yield was found in H_9_ over four consecutive crop growing seasons. Combined analysis over four seasons showed that studied variables together explained 85% of total variation in dependent structure (grain yield) obtained by Principal component analysis. This significant finding is the best example of phenotypic stability of grain yield in H_9_ and made it best fitted for grain yield under drought stress scenario. Detailed genetic analysis of H_9_ will help us to identify significant loci and alleles that made H_9_ the best fitted and it could serve as a potential source to generate novel transgressive levels of tolerance for drought stress in arid/semiarid regions.

## Introduction

Drought is a major constraint in agriculture system, significantly causes grain yield losses worldwide (Boyer, 1982) in maize crop. Water availability has become a serious issue in many parts of the world, both in developed and developing countries; due to un-even land, poor drainage system, scarcity of water channels persist, therefore, the development of crop cultivars with less water use is a common interest for breeders (Nafziger et al., 1991). In context of evolution, living organism choose different strategies to survive under environmental stress, for example, phenotypic plasticity (evolve new traits or modify existing traits with the appearance of new phenotypes) and phenotypic stability (stability of existing traits under particular stress), however, the general purpose is to be acclimatized under that environment for better survival (DeWitt and Langerhans, 2004).

Maize among cereals ranked at third number after wheat and rice worldwide (Ali F. et al., 2014). Over 80% of total global agricultural land is rain-fed (Berzsenyi et al., 2006) thus the development of genotypes that survive better in water scarce condition is the need of hour. Phenotypic stability of the traits in maize hybrids is the best way to measure the genetic variability (Chavan et al., 2015) by overwhelming the two most common variables in field: soil heterogeneity and environment (Khorasani et al., 2011; Ali et al., 2014a,c, 2015a). Therefore, multivariate analysis (Ashmawy, 2003; El-Badawy and Mehasen, 2011) displays a better idea of the underlying latent factors and an interface between individual genotype and variable. Other than this, genetic components, like genotypic correlation between traits, broad sense heritability and genetic advance could help us to understand the contributed genetic variation in population for better selection process. For a plant breeder, the major task is to reduce the error effects and to evaluate the total variation contributed by each trait. The stability in grain yield over multiple seasons is of quiet interest if we want to tackle the abiotic constraints, such as water scarce conditions (drought stress). Phenotypic stability of traits over multiple seasons could help us to identify the genes that inherit to next progenies, to achieve the desired product in next seasons.

The objectives of this study were (i) to evaluate maize hybrids in four consecutive crop growing seasons to measure the phenotypic stability of traits by assessing genotypic and phenotypic correlations, ${\text{h}}_{\text{b}.\text{s}}^{2}$, and genetic advance (ii) Identification of the most promising hybrid under drought stress condition and dissection of stable traits in winner genotype for possible use in future breeding program by applying QTL mapping, transcriptomics and genomic approaches.

## Materials and methods

### Irrigation regimes (an efficient way to develop the well-watered and drought stress conditions in field trials)

Irrigation regimes; drought stress and well-watered conditions were used as treatments in current study, to evaluate the performance of hybrids for grain yield. Normally, we applied first irrigation, 3 weeks after sowing. Here after this, we established the plots for drought stress and control conditions, for control plants we applied irrigation every week, and increased up-to two times a week during hot season May to July, considering rainfall also. To establish drought stress, we applied two irrigations fortnightly through-out each crop growing season (for experiment 2,3,4,5, see below) to apply and maintain drought stress conditions. Here we will only focus on drought stress experiment and the data has been provided for that only.

### Fertilizer (crop nutrients)

Leaf litter was added 1 month before seedbed preparation and mixed well by plowing at each experimental site. At sowing we added nitrogen 100 kg/ha, phosphorous 30 kg/ha and potassium 30 kg/ha and did plowing four times followed by planking each time.

### Genetic material (experiment 1)

Four inbred lines: two drought sensitive (PBG1 and PBG2) and two drought tolerant (PBG3 and PBG4) were selected from maize breeding program, Department of Plant Breeding and Genetics, University of Agriculture, Faisalabad-Pakistan. These lines were grown during March to June 2010–2011 and intercrossed in complete diallel fashion to develop twelve single cross maize hybrids (F_1_): H_1_ (PBG1 × PBG2), H_2_ (PBG1 × PBG3), H_3_ (PBG1 × PBG4), H_4_ (PBG2 × PBG1), H_5_ (PBG2 × PBG3), H_6_ (PBG2 × PBG4), H_7_ (PBG3× PBG1), H_8_ (PBG3× PBG2), H_9_ (PBG3× PBG4), H_10_ (PBG4 × PBG1), H_11_ (PBG4 × PBG2), H_12_ (PBG4 × PBG3). A randomized complete block design (RCBD) was used and the plot size was 3 × 3.3 m. Row-to-row and plant-to-plant distances were 75 and 15 cm, respectively, each row had 20 plants. Seed sowing was done using dibbler. Two seeds/hill of each parental line were sown and after 18 days thinned up to one healthy plant/hill. Howing, mowing, irrigation, and weeding (all agronomic practices) were done throughout the crop growing season. We did random sampling of 15 plants/plot of each genotype to measure the following traits: Ch.C (chlorophyll content), CW (cob weight), CL (cob length), CD (cob diameter), CPP (cob per plant), NGRC (number of grain rows per cob), SW (100 seed weight), NGPC (number of grains per cob), TDM (total dry matter), OC (oil content of grain), PC (protein content of grain), FSW (fresh stem weight), FLW (fresh leaf weight), FLWSR (fresh leaf weight to stem weight ratio), LA (leaf area), nlp (number of leaves per plant), PH (plant height), SD (stem diameter). Grain yield was recorded in kg per hectare. The moisture level of grains was adjusted (14–15.5%) to record grain yield. Soil was clay loam and the pH 7.85 was found.

### Experiment 2,3,4,5 (evaluation of F_1_ hybrids over four consecutive crop growing seasons for phenotypic stability of grain yield and its attributing traits under water scarce conditions)

Twelve single cross maize hybrids were evaluated from March to June 2011 (experiment 2), July to October 2011 (experiment 3), March to June 2012 (experiment 4) and July to October 2012 (experiment 5) in experimental research area of Department of Plant Breeding and Genetics, University of Agriculture, Faisalabad-Pakistan (31°26′ N, 73°06′ E). All the experiments planned according to experiment 1, followed by necessary agronomic practices. Same traits were studied as discussed in experiment 1.

### Crop growth and phenology [parental lines (2010) and F_1_ hybrids (2011–2012) were evaluated under drought stress in field conditions]

The dates of seedling emergence (when 50% of the plants emerged out of the soil surface and became visible), silking interval (when 50% of the plants appeared the visible silks in the field) and physiological maturity of plants (when 50% of the plants showed black layer formation in the grains from the mid portion of ear) for F_1_ hybrids were recorded during four crop growing seasons (2011–2012).

### Plant biomass

At physiological maturity; to estimate the plant density in plot, we counted the plants and divided the plant population by soil area. Central row plants were taken to reduce the treatment effects (irrigation, fertilizer application, hoeing etc.). Plant height was measured with measuring tape (least count ± 0.1 cm) placing it at the bottom portion of a plant up to the shoot. Plants then cut at basal level (ground) to record fresh leaves weight (g) and fresh stem weight (g), using electronic balance (OHAUS-GT4000, USA). Fresh leaves to stem weight ratio was obtained by dividing leaf weight to stem weight. The Digital Vernier Caliper (VMR/SHRZ) with least count (± 0.01 cm) was used to measure the stem diameter, the average value was taken by measuring the stem diameter at basal, middle and top portions. Green leaf area per plant was recorded according to formula L × W × 0.75 (Stewart and Dwyer, 1999). Leaf length and leaf width was recorded with measuring tape (least count ± 0.1 cm).

### Chlorophyll content, grain yield, and quality parameters

Chlorophyll content was measured at seedling, tasseling, booting, ear formation and grain filling stages by SPAD-502 chlorophyll meter (SPAD, soil plant analysis development) weekly and we took the average of all. The protein contents of maize seeds (ten seeds/genotype) were measured by absorbance assay (280 nm) and oil contents were measured, using technique introduced by Matthäus and Brühl (2001).

### Statistical analysis

#### ANOVA, DMRT

Two-way analysis of variance (ANOVA) was performed using the info-Genstat (12th edition) software for each crop growing season (experiment 2,3,4,5) as shown in (Supplementary Tables S1b, S2b, S3b, S4b). We used ANOVA to test the effect of drought stress, environment and their interaction on key traits, such as grain yield and its attributing traits. Duncan's multiple range test (DMRT) was used to compare the genotypic differences under drought stress (Supplementary Tables S1a, S2a, S3a, S4a).

#### Genetic components and correlation

Genetic components (genotypic variance, phenotypic variance, environmental variance, broad sense heritability and genetic advance) were determined in experiment 2–5 see Supplementary Tables S1c, S2c, S3c, S4c. We performed simple correlation analysis for experiment 2, 3, 4, and 5 (see Supplementary Tables S1d, S2d, S3d, S4d) to assess the magnitude of association among under studied traits.

#### Combined genotypic and phenotypic correlation

Combined genotypic and phenotypic correlations were calculated according to Falconer and Mackay (1996). The grand mean values were used to determine the phenotypic correlations as shown in (**Table 2**).

$$r_{AB}=\frac{\sum_{ij-1}^{n}\left(a_{ij}-{\bar{a}}_{ij})(b_{ij}={\bar{b}}_{ij}\right)}{\sqrt{\sum_{ij-1}^{n}{\left(a_{ij}-{\bar{a}}_{ij}\right)}^{2}\sum_{ij-1}^{n}{\left(b_{ij}-{\bar{b}}_{ij}\right)}^{2}}}fori\neq j,$$

Based on the phenotypic correlation between two traits or the same trait estimated in different environments: (Falconer and Mackay, 1996) we used the following method to determine the genotypic correlation (Table 1).

$$\begin{array}{l} r_{g}=\frac{r_{p}-e_{1}e_{2}r_{e}}{h_{1}h_{2}}\\ \;r_{e}=\frac{\sigma_{e12}^{2}}{\sigma_{e1}^{2}\sigma_{e2}^{2}} \end{array}$$

where *r*_*p*_ is the phenotypic correlation between traits 1 and 2 and *h*_1_ and *h*_2_ are the broad-sense heritability of trait 1 and 2, respectively. e_1_ and e_2_ are the environmental variance of trait 1 and 2, respectively and *r*_*e*_ is the environmental correlation between trait 1 and 2. The environmental correlation is not 0. Thus, the genetic correlation is a function of the phenotypic and environmental correlation as well as their heritability. The genotypic correlation was used as a matrix in path coefficient analysis (**Figure 4**) to determine the direct and indirect effect of yield and its attributing traits.

**Table 1:** Combined genotypic correlation coefficients (four variance matrices were used) for grain yield and its attributing traits (see Materials and Methods for traits description).

	**Ch.c**	**CW**	**CL**	**CD**	**CPP**	**NGRC**	**100 SW**	**NGPC**	**TDM**	**OC**	**PC**	**FLWSR**	**LA**	**nlp**	**PH**
CW	0.34														
CL	0.25	−0.61^**^													
CD	0.03	−0.09	−0.6^*^												
CPP	0.63^*^	−0.84^*^	−0.31	−1.12											
NGRC	0.94^**^	−0.01	−0.47	−0.56^**^	0.45										
SW	0.18	−0.2	−0.37	−0.13	0.04	0.37									
NGPC	0.51^*^	0.21	−0.3	−0.03	0.28	0.57	0.82^*^								
TDM	0.43	0.29	0.1	−0.23	0.02	0.07	0.22	0.35							
OC	0.33	0.68^*^	0.29	−0.24	0.15	0.76^**^	0.36	0.33	0.43						
PC	0.33	0.14	−0.07	−0.32	0.43	−0.36	0.73	0.03	0.51^**^	0.64^**^					
FLWSR	−0.01	0.46	0.41	−0.02	0.12	0.46	−0.25	0.1	−0.07	0.93^*^	0.03				
LA	0.23	0.43	−0.01	−0.04	0.14	0.56	0.68^**^	0.25	0.47	0.18	0.84^*^	0.08			
LP	−0.13	0.39	0.16	−0.43	0.44	0.39	0.79^*^	0.36	−0.25	0.59^**^	0.48	0.03	0.36		
PH	−0.02	0.62^*^	0.45	−0.44	0.27	0.36	0.97^*^	0.52^**^	0.53^**^	0.22	0.96^*^	0.09	0.68^**^	0.07	
GY	0.345^*^	−0.25	0.03	0.63^*^	0.98^*^	0.188^**^	0.51^*^	0.43^**^	0.32	0.33	−0.009	−0.019	−0.20	−0.38	−0.18

Values having

(*) *are significant at (p < 0.05)*.

Values having

(**) *are significant at (p < 0.01)*.

#### Multivariate analysis

To assess the overall variation attributed by yield attributing traits in hybrids, we performed principal component analyses (The Proc Mixed SAS version 9.1; SAS Institute, 2001) and construct bi-plot (combined data over four seasons used for each trait).

## Results

### Experiment 1 (development of successful intercrosses between drought tolerant and sensitive maize inbred lines in field conditions: March to June 2010)

**Figure 1:**
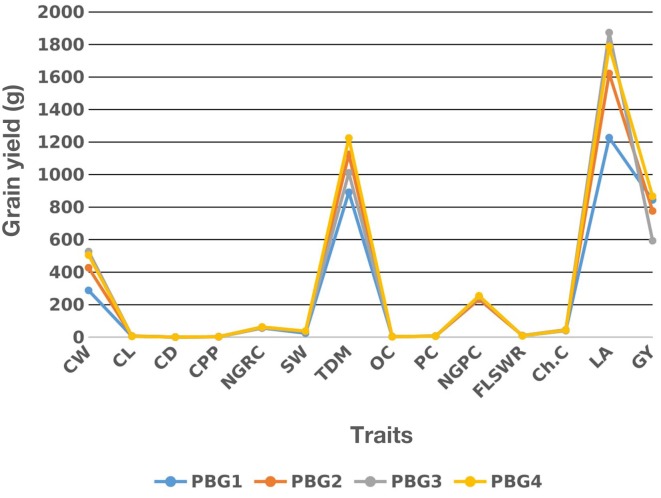
Mean performance of two drought sensitive (PBG1 and PBG2) and two drought tolerant (PBG3 and PBG4) maize inbred lines for grain yield and its attributing traits, see Section Materials and Methods for traits description.

Two drought sensitive (PBG1 and PBG2) and two drought tolerant (PBG3 and PBG4) maize inbred lines were intercrossed to develop F_1_ hybrids. Among these lines PBG4 performed better for grain yield followed by PBG3, PBG2, and PBG1 as shown in (Figure 1). The analysis of variance had significant (*P* < 0.05) results for under studied traits in these lines (data not shown).

### Experiment 2,3,4,5 (F_1_ hybrids under drought stress during, 2011–2012)

**Figure 2:**
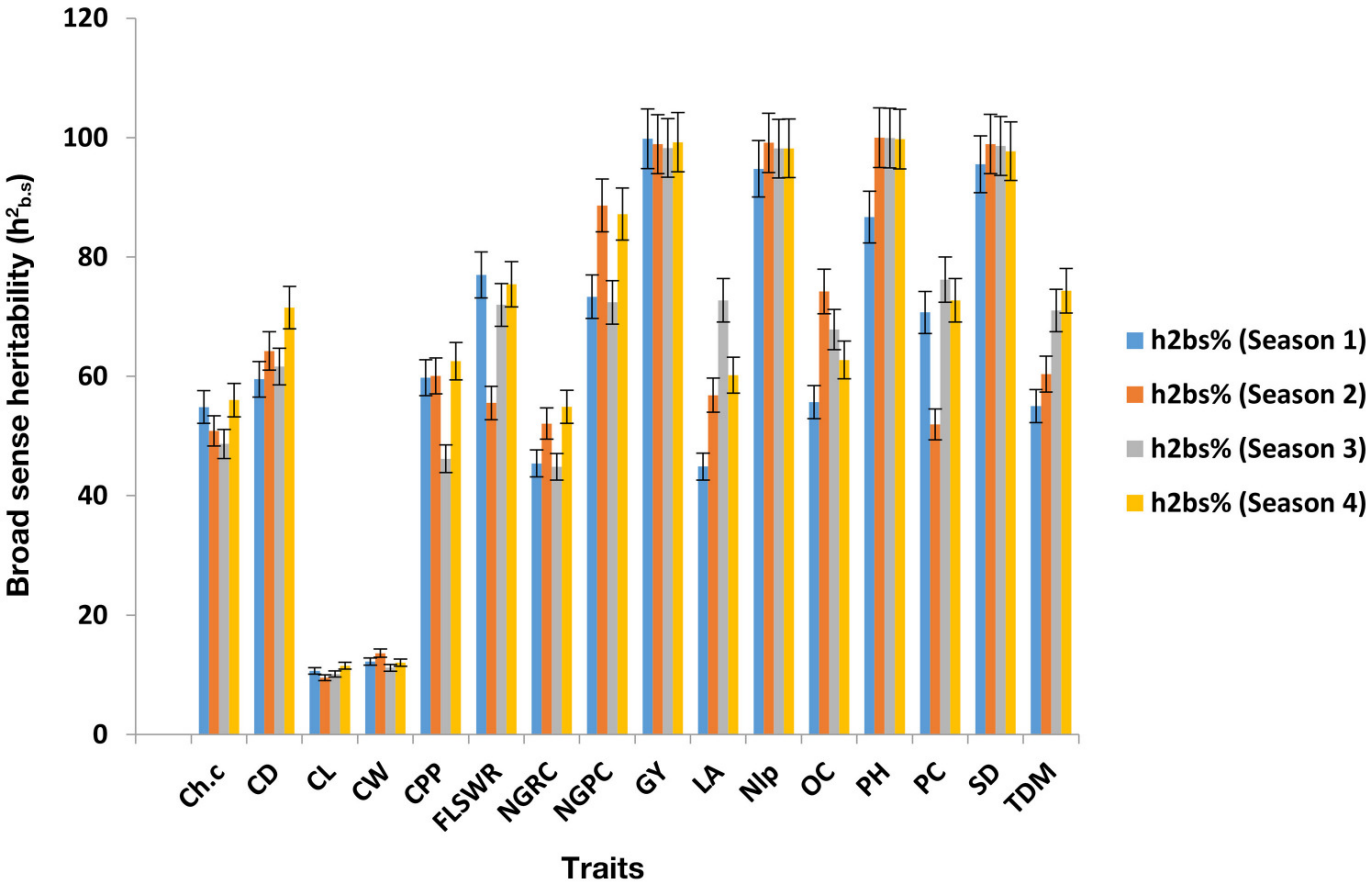
Broad sense heritability (${\text{h}}_{\text{b}.\text{s}}^{2}$) estimates of under study traits in H_9_. The error bars are Standard errors (±).

**Figure 3:**
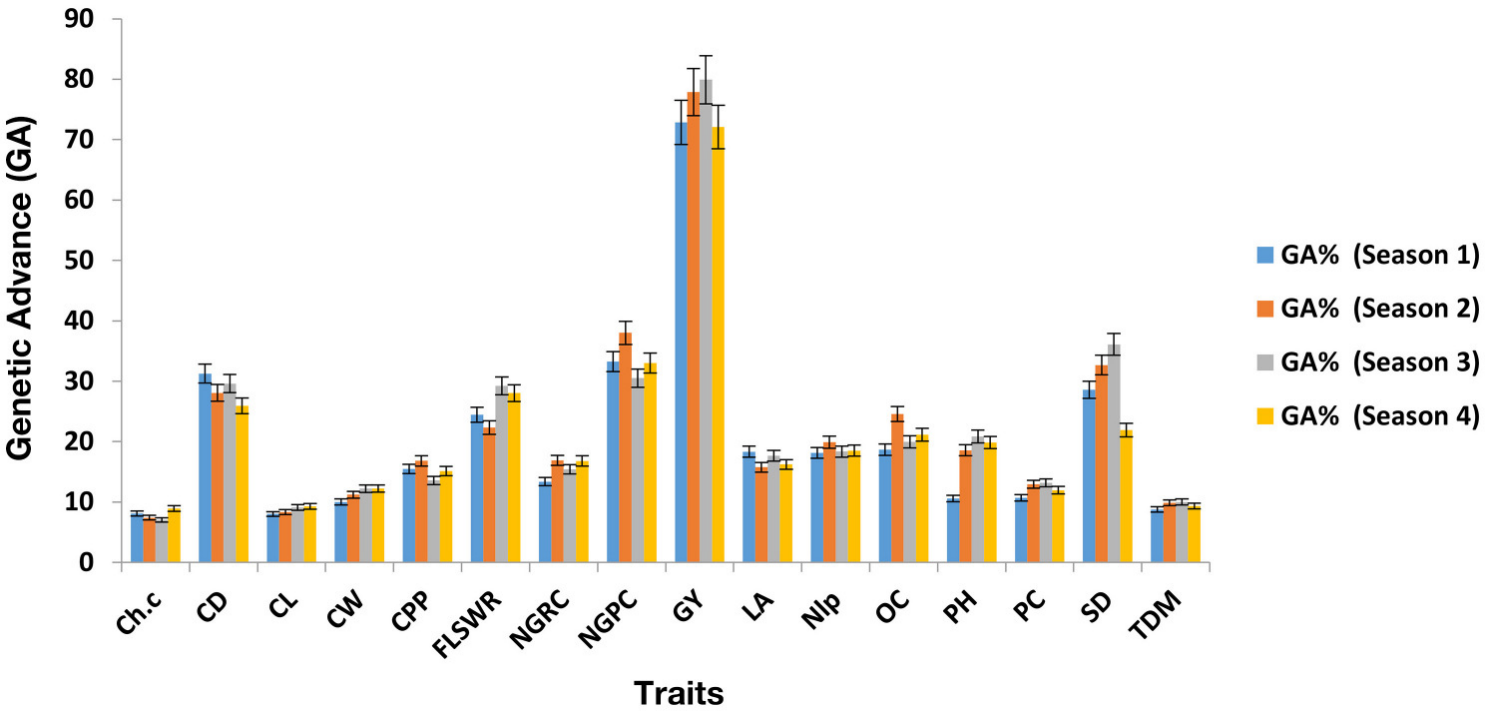
Genetic advance (GA) of under study traits in H_9_ (potential maize hybrid). The error bars are Standard errors (±).

Mean values comparison (Supplementary Table S1a, S2a, S3a, S4a) showed that hybrid H_9_ (PBG3 × PBG4) performed the best for grain yield in four seasons consecutively. ANOVA of under-studied traits found to be significant (*P* < 0.05) as shown in (Supplementary Tables S1b, S2b, S3b, S4b). Genetic components (Supplementary Table S1c, S2c, S3c, S4c) were also found to be significant in each crop growing season. Heritability and genetic advance are given (**Table 4**) for each trait, measured over four seasons and found to be significant for grain yield, chlorophyll content, plant height and total dry matter in each crop growing seasons in hybrid H_9_ as shown (Figures 2, 3). To assess the magnitude of association for grain yield and its attributing traits in each crop growing seasons, we performed pearson's correlation analysis (Table S1d, S2d, S3d, S4d) and found that chlorophyll content, plant height, total dry matter, cob diameter, cob length, cob weight, number of grain rows per cob, fresh leaf weight to stem weight ratio and grain yield are closely related to each other.

### Genotypic and phenotypic correlations (combined analysis for four seasons)

We combined the variances of each trait, measured in four crop growing seasons and used them in combined analysis to find out the genotypic and phenotypic correlations. Genotypic correlation coefficients (Table 1) showed that chlorophyll contents, plant height, total dry matter, cobs per plant, protein contents and oil contents had positive and significant correlation (*P* < 0.01) to grain yield per plant under water stress conditions. The phenotypic correlation coefficients (*P* < 0.01) between each of two traits was examined (Table 2), showed significant and positive association of chlorophyll contents, cobs per plant, grain rows per cob, grains per cob, total dry matter, oil content, protein content, fresh leaves weight to stem weight ratio, plant height and leaf area to grain yield/plant.

**Table 2:** Combined phenotypic correlation coefficients (four variance matrices were combined for each trait) among grain yield and its attributing characters (see Materials and Methods for traits description).

	**Ch.c**	**CW**	**CL**	**CD**	**CPP**	**NGRC**	**SW**	**NGPC**	**TDM**	**OC**	**PC**	**FLWSR**	**LA**	**LP**	**PH**
CW	0.37														
CL	0.21	0.16													
CD	0.19	0.57	0.46												
CPP	0.14	0.58	0.14	0.21											
NGRC	0.13	0.51	0.36	0.62^**^	−0.02										
SW	0.44	0.66^**^	0.21	0.39	0.14	0.39									
NGPC	0.25	0.41	−0.09	0.38	−0.19	0.45	0.63^**^								
TDM	0.33	0.23	0.05	0.11	0.22	0.31	0.49	0.33							
OC	0.38	0.21	0.25	0.46	−0.18	0.45	0.61^**^	0.69^**^	0.14						
PC	0.26	0.49	0.05	0.51^**^	−0.17	0.42	0.41	0.34	0.26	0.44					
FLWSR	0.28	−0.01	0.24	−0.01	−0.05	0.26	0.08	0.08	0.33^*^	0.15	0.18				
LA	0.79^*^	−0.38	−0.06	−0.1	−0.08	−0.19	−0.28	−0.03	−0.1	−0.25	−0.32	−0.31			
LP	−0.27	−0.13	−0.25	−0.22	−0.11	0.02	0.13	−0.04	0.21	−0.04	0.11	−0.32	0.45		
PH	0.33^*^	−0.03	0.53^**^	−0.05	−0.12	0.09	0.26	0.46	0.04	0.25	0.28	−0.25	0.22	0.47	
GY	0.86^*^	0.26	−0.03	−0.46	0.82^*^	0.77^*^	0.59^**^	0.78^*^	0.91^*^	0.74^*^	0.81^*^	0.61^**^	0.85^**^	−0.19	0.86^*^

Values having

(*) *are significant at (p < 0.05)*.

Values having

(**) *are significant at (p < 0.01)*.

### Path coefficient analysis: combined analysis for four seasons

**Figure 4:**
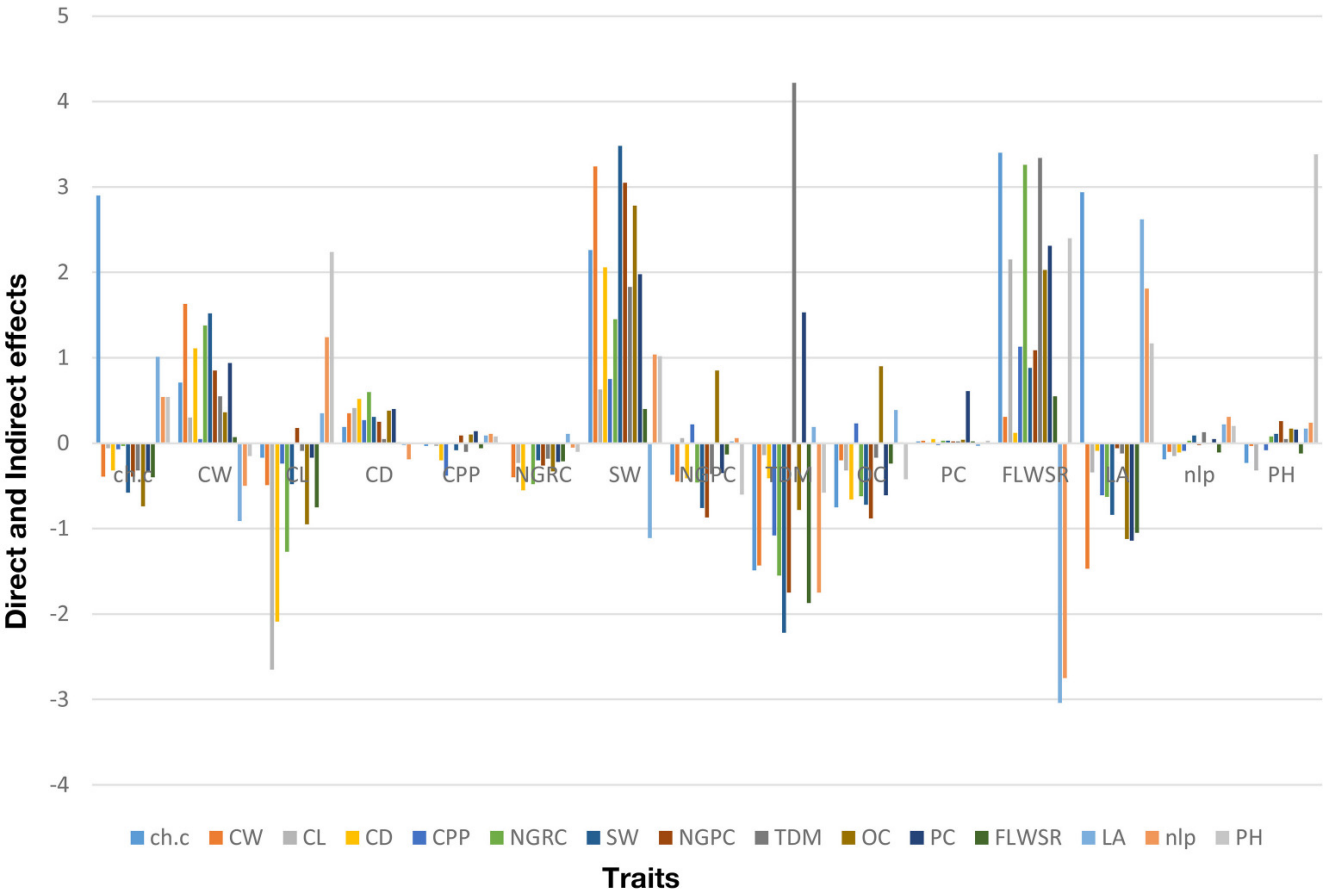
Percentage contribution of grain yield and its attributing traits based on path coefficient analysis for four consecutive crop growing seasons (see Section Materials and Methods for traits description).

The genotypic correlation was used as a matrix to calculate the direct and indirect effect of each trait on grain yield under droughts stress conditions. The percentage contribution of each trait is given (+4.0 to −2.5) as shown in (Table 3; Figure 4), calculated over four crop growing seasons. The results (Figure 4) shows that total dry matter had the highest direct effect on grain yield per pant while indirect effect via stem diameter and protein content were also significant. Chlorophyll content had the significant direct effect and indirectly contributed to grain yield per plant via cob length, cob weight and leaf area. The direct effect of cob weight was significant and indirect contribution to grain yield per plant was also significant via other traits except cob length, number of leaves per plant and plant height. Cob diameter had the negative indirect effect only through number of leaves per plant. 100 seed weight interestingly had the significant direct effect, while its indirect effect were almost contributing to grain yield except one trait (leaf area). Fresh leaf weight to stem weight ratio also contributed significantly for direct and indirect effects and put a major contribution to grain yield under drought stress. Plant height had the significant direct effect but its indirect effects were negligible (Table 3). These results suggest that the traits which had strong direct effect at genotypic level to grain yield, should be selected in breeding program to develop climate resilient maize hybrids under water scarce conditions.

**Table 3:** Direct and indirect effects of each yield attributing trait [Diagonal (bold) is the direct effect while others are indirect effects], combined analysis was done for each trait, evaluated over four crop growing season.

	**Ch.c**	**CW**	**CL**	**CD**	**CPP**	**NGRC**	**SW**	**NGPC**	**TDM**	**OC**	**PC**	**FLWSR**	**nlp**	**LP**	**PH**	**rg**
Ch.c	**2.9**	0.71	−0.17	0.19	−0.03	−0.01	2.26	−0.37	−1.49	−0.75	0.02	3.4	2.94	−0.19	−0.23	0.3458
CW	−0.39	**1.63**	−0.49	0.35	−0.01	−0.4	3.24	−0.45	−1.43	−0.2	0.03	0.31	−1.47	−0.1	−0.03	0.2560
CL	−0.06	0.3	−**2.65**	0.41	−0.03	−0.23	0.63	0.06	−0.14	−0.32	0	2.15	−0.34	−0.15	−0.32	0.0385
CD	−0.32	1.11	−2.09	**0.52**	−0.2	−0.55	2.06	−0.41	−0.41	−0.66	0.05	0.12	−0.09	−0.11	0	0.6306
CPP	−0.07	0.05	−0.24	0.27	−**0.38**	−0.01	0.75	0.22	−1.08	0.23	−0.02	1.13	−0.61	−0.09	−0.08	0.9800
NGRC	−0.03	1.38	−1.27	0.6	0	−**0.48**	1.45	−0.46	−1.55	−0.62	0.03	3.26	−0.63	0.03	0.08	0.1880
SW	−0.58	1.52	−0.48	0.31	−0.08	−0.2	**3.48**	−0.76	−2.22	−0.72	0.03	0.88	−0.84	0.09	0.11	0.5107
NGPC	−0.39	0.85	0.18	0.25	0.09	−0.26	3.05	−**0.87**	−1.75	−0.88	0.02	1.09	−0.06	−0.02	0.26	0.4324
TDM	−0.32	0.55	−0.09	0.05	−0.1	−0.18	1.83	−0.36	**4.22**	−0.17	0.02	3.34	−0.12	0.13	0.05	0.3275
OC	−0.74	0.36	−0.95	0.38	0.1	−0.33	2.78	0.85	−0.78	**0.9**	0.04	2.03	−1.12	0	0.17	0.3323
PC	−0.34	0.94	−0.17	0.4	0.14	−0.22	1.98	−0.35	1.53	−0.61	**0.61**	2.31	−1.14	0.05	0.16	−0.0097
FLWSR	−0.4	0.07	−0.75	0.01	−0.06	−0.21	0.4	−0.13	−1.87	−0.24	0.02	**0.55**	−1.05	−0.11	−0.12	−0.0198
LA	1.01	−0.91	0.35	−0.02	0.09	0.11	−1.11	0.02	0.19	0.39	−0.03	−3.04	**2.62**	0.22	0.17	−0.2096
LP	0.54	−0.5	1.24	−0.19	0.11	−0.05	1.04	0.06	−1.75	0.01	0.01	−2.75	1.81	**0.31**	0.24	−0.3855
PH	0.54	−0.15	2.24	−0.01	0.08	−0.1	1.02	−0.6	−0.58	−0.42	0.03	2.4	1.17	0.2	**3.38**	−0.1839

*We used combined genotypic correlation (Table 2) value of each trait as a matrix to find out the direct and indirect effects (see Materials and Methods for traits description)*.

### Genotype × environment interaction (biplot analysis)

**Figure 5:**
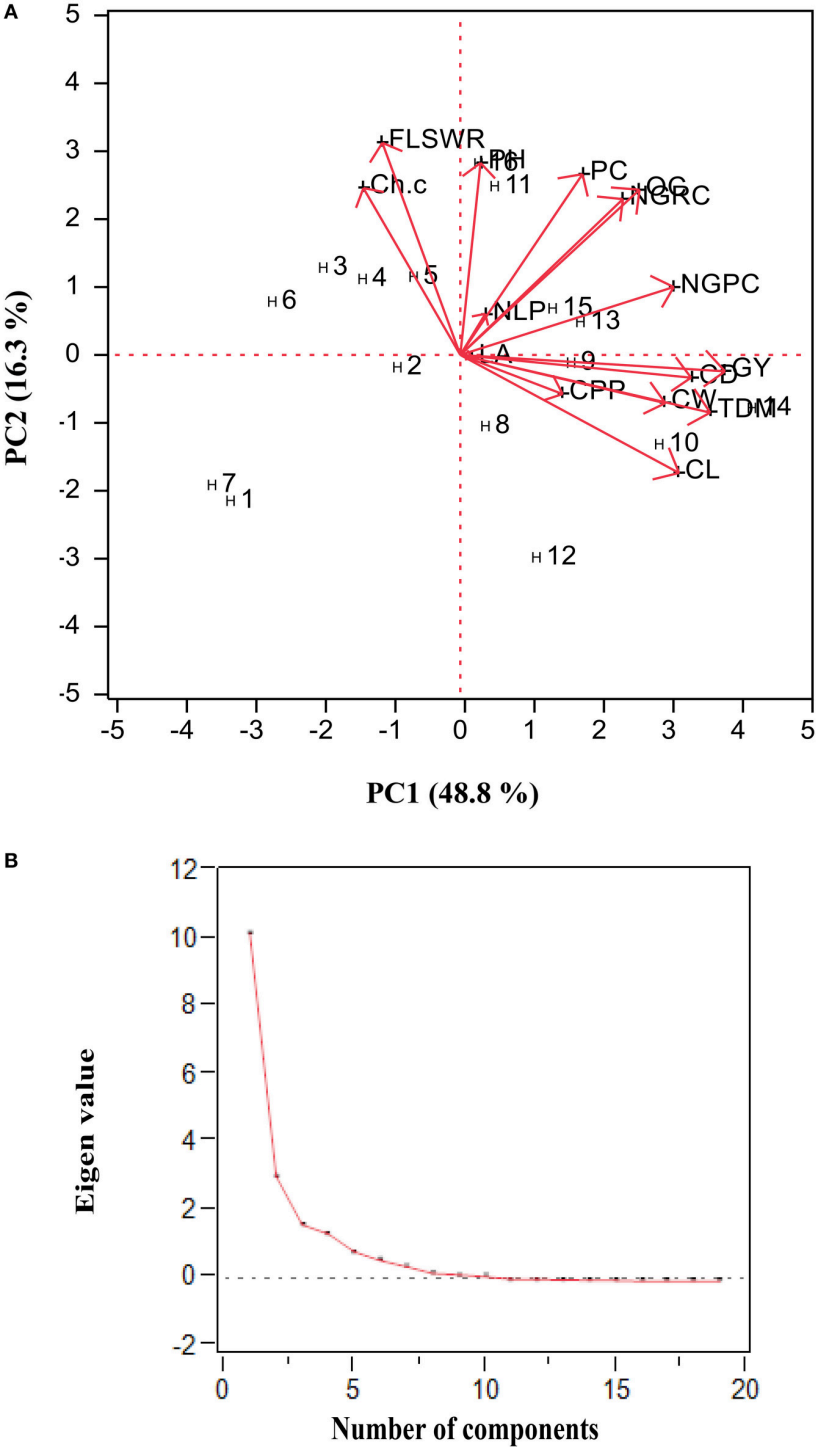
**(A)** Biplot analysis (Hybrids × traits interaction) based upon of four crop growing seasons data (2010–2011 and 2011–2012) of under study maize hybrids (see Section Materials and Methods for hybrid codes and traits description). **(B)** Scree plot of principal component analysis: PC_1_ (10.2), PC_2_ (3.1), PC_3_ (1.6), and PC_4_ (1.3) which together explained 85.5% of total variation in the dependent structure.

Combined data (collected over four seasons for each trait) was used to develop biplot (genotypes × traits association) as shown in (Figure 5A). Based on principal component analysis we generated biplot (graphical display of variables and hybrids) and obtained four principal components had eigen values > 1 (*P* < 0.001): PC1 (10.2), PC2 (3.1), PC3 (1.6), and PC4 (1.3)], which together explained 85.5% of total variation (Figure 5B). The trait vectors (plant height, chlorophyll contents, total dry matter, had small angles with grain yield were the most significant and positively correlated to H_9_ (Figure 5A), while other traits were least contributing to grain yield.

## Discussion

To breed the maize cultivars for grain yield stability under water scarce conditions (drought stress) is a serious threat to food security. Can we breed for plasticity in the key agronomic traits (Question 5; Nicotra et al., 2010) to improve the stability in grain yield and its attributing traits, is a major concern for plant breeders. In context of this, maize breeding for yield stability has become an important task to evaluate the hybrids, lines, and cultivars in a single and/or over a range of environments/multiple seasons. The phenotypic stability of yield and its related traits in a single location over multiple crop growing seasons could help us to identify the genes that could be used as a potential source in maize breeding to develop drought tolerant cultivars. In the present study we evaluated 12 single cross maize hybrids in a single location over 4 crop growing seasons (2010–2012), and found hybrid H_9_ a potential candidate genotype that showed phenotypic stability for grain yield under water scarce conditions. The phenotypic stability in grain yield and its related traits showed that genotypic responses were more in each growing season. Finding these interesting results, we observed in H_9_, the broad sense heritability (${\text{h}}_{\text{b}.\text{s}}^{2}$) and genetic advance in each crop growing season and found higher ${\text{h}}_{\text{b}.\text{s}}^{2}$ and genetic advance for grain yield, plant height, chlorophyll content and total dry matter as shown (Figures 2, 3 and Table 4). The higher values of genetic advance and their phenotypic stability (Ali et al., 2013, 2014d) in each crop season shows that H_9_ is potential genome to develop maize genotypes that could breed in an environment where water supply is limited (Opitz et al., 2016).

**Table 4:** Comparative analysis: genetic advance (GA %) and broad sense heritability (${\text{h}}_{\text{b}.\text{s}}^{2}$ %) of under studied traits in H_9_ maize hybrid evaluated for four consecutive crop growing seasons i.e., experiments 2–5 (see Section Materials and Methods for traits description) under drought stress.

	**Genetic Advance (GA%)**				**Broad Sense Heritability (**${\text{h}}_{\text{b}.\text{s}}^{2}$**%)**			
**Traits**	**GA%**	**GA%**	**GA%**	**GA%**	**${\text{h}}_{\text{b}.\text{s}}^{2}$%**	**${\text{h}}_{\text{b}.\text{s}}^{2}$%**	**${\text{h}}_{\text{b}.\text{s}}^{2}$%**	**${\text{h}}_{\text{b}.\text{s}}^{2}$%**
	**(Season 1)**	**(Season 2)**	**(Season 3)**	**(Season 4)**	**(Season 1)**	**(Season 2)**	**(Season 3)**	**(Season 4)**
Ch.c	8.085	7.405	7.022	8.918	54.86	50.862	48.676	56.028
CD	31.257	28.071	29.611	25.950	59.52	64.257	61.650	71.513
CL	8.008	8.348	9.108	9.265	10.658	9.499	10.134	11.500
CW	10.013	11.194	12.217	12.224	12.201	13.626	11.178	12.045
CPP	15.482	16.806	13.571	15.106	59.78	60.095	46.186	62.533
FLSWR	24.459	22.301	29.220	28.031	76.99	55.532	71.979	75.43
NGRC	13.36	16.896	15.416	16.796	45.41	52.090	44.836	54.902
NGPC	33.237	37.996	30.500	33.005	73.35	88.640	72.394	87.181
GY	72.855	77.850	79.914	72.095	99.79	98.904	98.250	99.226
LA	18.335	15.758	17.674	16.219	44.88	56.851	72.725	60.193
Nlp	18.126	19.908	18.356	18.510	94.77	99.126	98.170	98.195
OC	18.66	24.575	19.955	21.134	55.67	74.227	67.846	62.755
PH	10.595	18.560	20.847	19.856	86.68	99.987	99.957	99.745
PC	10.673	12.913	13.159	11.960	70.71	51.947	76.215	72.744
SD	28.574	32.665	36.086	21.905	95.54	98.935	98.585	97.727
TDM	8.771	9.863	9.991	9.340	55.02	60.386	71.049	74.339

In maize breeding, several authors have quantified the relationship between grain yield and its attributing morphological traits (Imtiaz et al., 2009; Zare et al., 2011; Ali et al., 2014b; Khan et al., 2014) but in a single environment and/or season. Similar investigations were reported by Mahmood et al. (2004) and Ali et al. (2011a,b), they observed that leaf area, plant height, 100 seed weight, cobs per plant and grain yield per plant are closely associated traits, but all these findings were limited to a single environment and/or season. In the present study, significant genetic correlations (Table 1) were found between grain yield and traits, such as plant height, total dry matter, chlorophyll content, leaf area, stem weight, seed weight, cob length and weight, seed quality parameters (protein and oil). The importance of morphological traits, such as leaf area index (Amanullah et al., 2014) has been investigated in maize hybrids and stated to be a main component for vigorous photosynthetic rate in maize hybrids, also reported by Cirilo et al. (2009) and Stewart and Dwyer (1999). Similarly, chlorophyll contents were measured from seedling to tasseling, and reported to be a major contributor for vegetative growth rate Ding et al. (2007) and Perveen et al., 2013 in maize. Weber et al. (2012) also reported that plants which had more chlorophyll content drives photosynthetic machinery more frequently that may facilitate to increase grain yield.

Path coefficient analysis is an efficient tool for a plant breeder, to visualize the direct and indirect effect of a single trait on dependent structure (Ali et al., 2011a; El-Badawy and Mehasen, 2011) and could provide a better understanding about the selection process in breeding program, especially, when a large number of variables are involved (Beiragi et al., 2012). The traits which had the most positive direct effect are favorable to select in the breeding cycle (Rubino and Davis, 1990). The use of path analysis in previous findings is limited to its application in a single environment and/or season study, however, for combined data collected over multiple seasons (as we did in the present study), the use pf path analysis is not available. Generally, genotypic variances are used in the path coefficient matrix, therefore, the combined genotypic variances calculated over multiple seasons, could improve the power of analysis. The present study results (Figure 4) demonstrates that direct effect of total dry matter, chlorophyll content, plant height, 100 seed weight, cob diameter was significantly higher as compared to other traits, and also stable over multiple seasons to contribute genetic variation (Figures 2, 3).

Our results shows that,

1. Genotypic and phenotypic correlations (combined analysis) gave us the magnitude of association between traits (Phenotypic stability for traits association).
2. Path coefficient analysis was used to determine the direct and indirect effects to improve the process of selection (by assessing the phenotypic stability).
3. Broad sense heritability (${\text{h}}_{\text{b}.\text{s}}^{2}$) of traits shows how much is the genetic contribution.
4. Genetic advance tells us the performance of developed hybrids over parents (resistant and tolerant parents to drought stress), and H_9_ outperformed for grain yield in each season.

To assess the overall variation attributed by under study traits, we performed multivariate analysis. This approach is useful for a plant breeder, to select the traits for the most promising genotypes, saves time, to be more watchful and selective to handle a large amount of data as reported by Cirilo et al. (2009) and Ali et al. (2015b). The present study shows that chlorophyll content, cobs per plant, number of grains rows per cob, total dry matter and cob diameter were the most significant traits that contribute to grain yield under drought stress conditions. Classification of agronomic and morphological traits using multivariate analysis approach is useful to save money and time in crop improvement program. On the basis of present study results, it is recommended that hybrid H_9_ is a potential genotype that had high grain yield in arid/semi-arid region under drought stress in field conditions over multiple seasons. The stability of traits like grain yield, plant height, chlorophyll content and total dry matter is of great interest that favors the future genetic analysis in H_9_ to find out the QTLs associated with traits stability. Moreover, the transcriptomics studies could help us to understand more about the gene regulatory components in H_9_ that could be dissected for their possible use in maize breeding program for drought tolerance.

## Author contributions

FA conducted research under the supervision of MA and write up initial draft of manuscript. QA, NK, and FA carried out statistical analysis and interpretation of data. NK make corrections in the final manuscript and edited for final version. All of the authors proof-read the manuscript and approve for publication.

### Conflict of interest statement

The authors declare that the research was conducted in the absence of any commercial or financial relationships that could be construed as a potential conflict of interest.
