# Supplementary material for: Phenotypic Stability of Zea mays Grain Yield and Its Attributing Traits under Drought Stress
Source: Front Plant Sci. 2017 Aug 22;8:1397. doi: 10.3389/fpls.2017.01397 (PMC5572255; doi:10.3389/fpls.2017.01397)
Supplement: Supplementary file 1 [file Table1.pdf]

Supplementary Tables

Supplementary Tables S1a,b,c,d (Data of crop growing season 1 i.e February 2010-2011).

Supplementary Tables S2a,b,c,d (Data of crop growing season 2 i.e August 2010-2011).

Supplementary Tables S3a,b,c,d (Data of crop growing season 3 i.e February 2011-2012).

Supplementary Tables S4a,b,c,d (Data of crop growing season 4 i.e August 2011-2012).

(Suppl. Table S1a). Duncan Multiple Range Test (DMRT) of under studies traits in twelve maize hybrids during crop growing season 1 (February 2010-2011) [see section materials and methods for hybrid codes and traits description].

Traits													
Ch.c	Hybrids	H9	H8	H5	H11	H1	H12	H6	H2	H4	H10	H7	H3
	DMRT Values	59.667	49.883	49.120	48.627	48.347	47.743	46.220	46.070	45.503	44.470	42.893	40.573
	Pair wise comparison	A	B	BC	BC	BC	BC	BC	BC	BC	BC	BC	C
C.D	Hybrids	H9	H11	H5	H12	H2	H10	H3	H4	H6	H7	H8	H1
	DMRT Values	2.3500	1.4167	1.3333	1.2633	1.2133	1.2000	1.2000	1.2000	1.2000	1.2000	1.1333	1.0867
	Pair wise comparison	A	B	B	B	B	B	B	B	B	B	B	B
C.L	Hybrids	H9	H1	H11	H12	H8	H5	H2	H6	H3	H7	H4	H10
	DMRT Values	8.7433	7.9333	7.6667	7.4000	7.3000	7.2000	7.1000	6.9000	6.8000	6.8000	6.7667	6.7000
	Pair wise comparison	A	AB	AB	AB	AB	AB	B	B	B	B	B	B
C.W	Hybrids	H9	H5	H6	H12	H8	H2	H3	H11	H1	H7	H4	H10
	DMRT Values	155.00	119.57	116.73	110.40	107.93	107.27	106.13	102.20	100.27	97.87	97.13	87.97
	Pair wise comparison	A	AB	AB	AB	AB	AB	B	B	B	B	B	B
CPP	Hybrids	H9	H11	H1	H3	H6	H10	H12	H2	H4	H7	H8	H5
	DMRT Values	3.6667	3.3333	3.0000	3.0000	3.0000	2.6667	2.6667	2.6667	2.6667	2.6667	2.6667	2.3333
	Pair wise comparison	A	A	A	A	A	A	A	A	A	A	A	A
FLSWR	Hybrids	H9	H12	H11	H5	H7	H10	H8	H4	H6	H3	H1	H2
	DMRT Values	5.1067	3.9767	3.7400	3.6633	3.5000	3.4167	3.3800	3.2800	3.2200	3.1000	3.0500	2.8700
	Pair wise comparison	A	B	BC	BCD	BCDE	CDE	CDEF	CDEF	DEF	EF	EF	F
GRPC	Hybrids	H9	H10	H11	H12	H3	H8	H2	H4	H5	H6	H1	H7
	DMRT Values	17.33	15.333	15.333	15.333	15.333	15.333	14.667	14.667	14.667	14.667	14.000	13.333
	Pair wise comparison	A	AB	AB	AB	AB	AB	B	B	B	B	B	B
NGPC	Hybrids	H9	H10	H12	H11	H8	H6	H7	H5	H4	H3	H2	H1
	DMRT Values	376.33	310.00	307.00	302.00	294.33	283.00	273.00	272.33	257.00	245.00	240.00	191.00
	Pair wise comparison	A	B	B	B	BC	BCD	BCD	BCD	CD	D	D	E
L.A	Hybrids	H9	H12	H11	H7	H4	H8	H5	H6	H3	H10	H2	H1
	DMRT Values		777.00	774.00	762.40	735.87	724.13	712.40	707.78	662.33	657.22	602.67	584.00

		826.67											
	Pair wise comparison	A	AB	AB	AB	AB	ABC	ABCD	ABCD	BCD	BCD	CD	D
Nlp	Hybrids	H9	H6	H1	H10	H3	H4	H5	H2	H8	H11	H12	H7
	DMRT Values	14.000	13.000	12.000	12.000	12.000	12.000	12.000	11.000	11.000	10.000	10.000	10.000
	Pair wise comparison	A	B	C	C	C	C	C	D	D	E	E	E
OC	Hybrids	H9	H10	H7	H12	H4	H5	H6	H8	H11	H2	H3	H1
	DMRT Values	4.9633	4.9367	4.5367	4.3933	4.3167	4.1033	4.0567	4.0367	3.8933	3.6533	3.5967	3.3567
	Pair wise comparison	A	A	AB	AB	ABC	ABC	ABC	ABC	BC	BC	BC	C
PH	Hybrids	H9	H6	H7	H3	H10	H4	H5	H12	H8	H1	H11	H2
	DMRT Values	276.67	249.67	249.33	248.00	241.73	240.67	237.67	236.30	233.47	228.00	221.13	214.67
	Pair wise comparison	A	B	B	B	BC	BC	CD	CD	CD	DE	EF	F
PC	Hybrids	H9	H12	H8	H11	H4	H5	H7	H10	H6	H2	H1	H3
	DMRT Values	9.2500	8.6467	7.9433	7.9033	7.8700	7.6967	7.6433	7.5867	7.5100	7.3700	7.2433	7.1033
	Pair wise comparison	A	A	B	B	B	BC	BC	BC	BC	BC	C	C
SD	Hybrids	H9	H4	H10	H3	H6	H1	H11	H12	H5	H7	H2	H8
	DMRT Values	0.8267	0.6900	0.6400	0.6200	0.6000	0.5800	0.5800	0.5600	0.5600	0.5600	0.5500	0.5433
	Pair wise comparison	A	B	C	CD	DE	EF	EF	FG	FG	FG	G	G
TDM	Hybrids	H9	H1	H6	H12	H11	H2	H3	H8	H4	H7	H10	H5
	DMRT Values	1505.0	1239.2	1222.7	1193.1	1192.8	1192.6	1191.4	1187.5	1183.0	1136.4	1050.1	1047.2
	Pair wise comparison	A	B	B	B	B	B	B	B	B	B	B	B
GY	Hybrids	H9	H12	H3	H11	H6	H8	H10	H4	H5	H1	H2	H7
	DMRT Values	1722.3	1477.0	1088.0	1052.0	888.0	870.0	867.0	789.0	596.0	582.0	528.0	488.0
	Pair wise comparison	A	B	C	D	E	E	E	F	G	G	H	I

Supplementary Table S1b: ANOVA (Analysis of variance) of all studied traits in twelve maize hybrids during crop growing season 1 (August 2010-2011) of grain yield and its attributing traits.

Randomized Complete Block AOV Table for Chlorophyll contents

**Source**	**DF**	**SS**	**MS**	**F**	**P**
repli	2	42.79	21.3953		
hybrids	11	733.09	66.6447	2.61	0.0002
Error	22	562.65	25.5750		
Total	35	1338.53			

Grand Mean 47.426 CV 10.66

Randomized Complete Block AOV Table for Cob diameter

**Source**	**DF**	**SS**	**MS**	**F**	**P**
repli	2	0.00054	0.00027		
hybrids	11	3.73843	0.33986	5.41	0.0004
Error	22	1.38126	0.06278		
Total	35	5.12023			

Grand Mean 1.3164 CV 19.03

Randomized Complete Block AOV Table for Cob length

**Source**	**DF**	**SS**	**MS**	**F**	**P**
repli	2	0.1276	0.06381		
hybrids	11	11.9286	1.08442	1.28	0.0002
Error	22	18.5765	0.84438		
Total	35	30.6327			

Grand Mean 7.2758 CV 12.63

Randomized Complete Block AOV Table for Cob weight

**Source**	**DF**	**SS**	**MS**	**F**	**P**
repli	2	53.2	26.577		
hybrids	11	9394.3	854.030	1.07	0.0007
Error	22	17600.3	800.015		
Total	35	27047.8			

Grand Mean 109.04 CV 25.94

Randomized Complete Block AOV Table for cobs per plant

Source	DF	SS	MS	F	P
repli	2	0.2222	0.11111		
hybrids	11	15.7778	1.4344	7.3295	0.0006
Error	22	4.3056	0.1957		
Total	35	20.3056			

Grand Mean 2.8611 CV 29.60

Randomized Complete Block AOV Table for fresh leaf to weight ratio

Source	DF	SS	MS	F	P
repli	2	1.7243	0.86214		
hybrids	11	11.3782	1.03438	11.03	0.0000
Error	22	2.0626	0.09375		
Total	35	15.1651			

Grand Mean 3.5253 CV 8.69

Randomized Complete Block AOV Table for grain rows per cob

Source	DF	SS	MS	F	P
repli	2	2.6667	1.33333		
hybrids	11	30.6667	2.78788	1.77	0.0001
Error	22	34.6667	1.57576		
Total	35	68.0000			

Grand Mean 15.000 CV 8.37

Randomized Complete Block AOV Table for grains per cob

Source	DF	SS	MS	F	P
repli	2	705.2	352.58		
hybrids	11	68950.7	6268.25	9.26	0.0000
Error	22	14894.8	677.04		
Total	35	84550.7			

Grand Mean 279.25 CV 9.32

Randomized Complete Block AOV Table for grain yield per plant

Source	DF	SS	MS	F	P
repli	2	636	318		
hybrids	11	4745767	431433	1487.13	0.0000
Error	22	6382	290		
Total	35	4752785			

Grand Mean 912.28 CV 1.87

Randomized Complete Block AOV Table for leaf area

Source	DF	SS	MS	F	P
repli	2	9393	4696.7		
hybrids	11	174817	15892.4	2.61	0.0020
Error	22	134132	6096.9		
Total	35	318342			

Grand Mean 710.54 CV 10.99

Randomized Complete Block AOV Table for leaves per plant

Source	DF	SS	MS	F	P
repli	2	0.1667	0.08333		
hybrids	11	50.7500	4.61364	55.36	0.0000
Error	22	1.8333	0.08333		
Total	35	52.7500			

Grand Mean 11.583 CV 2.49

Randomized Complete Block AOV Table for oil contents

Source	DF	SS	MS	F	P
repli	2	0.3923	0.19614		
hybrids	11	8.3656	0.76051	2.27	0.0044
Error	22	7.3859	0.33572		
Total	35	16.1438			

Grand Mean 4.1536 CV 13.95

Randomized Complete Block AOV Table for plant height

Source	DF	SS	MS	F	P
repli	2	80.71	40.356		

hybrids	11	8386.17	762.379	20.52	0.0000
Error	22	817.47	37.158		
Total	35	9284.35			

Grand Mean 239.77   CV 2.54

Randomized Complete Block AOV Table for protein contents

**Source**	**DF**	**SS**	**MS**	**F**	**P**
repli	2	0.2178	0.10889		
hybrids	11	11.9943	1.09039	8.24	0.0000
Error	22	2.9104	0.13229		
Total	35	15.1225			

Grand Mean 7.8139   CV 4.65

Randomized Complete Block AOV Table for stem diameter

**Source**	**DF**	**SS**	**MS**	**F**	**P**
repli	2	5.760E-32	2.880E-32		
hybrids	11	0.21534	0.01958	65.92	0.0000
Error	22	0.00653	2.970E-04		
Total	35	0.22187			

Grand Mean 0.6092   CV 2.83

Randomized Complete Block AOV Table for total dry matter

**Source**	**DF**	**SS**	**MS**	**F**	**P**
repli	2	42185	21092.7		
hybrids	11	435950	39631.9	1.95	0.0002
Error	22	447502	20341.0		
Total	35	925638			

Grand Mean 1195.1   CV 11.93

Table S1c. Genetic Components for under studied traits in H_9 maize hybrid during crop growing season 1 (February 2010-2011) under water stress environment.

Traits	M.S	G.M	GV	GCV %	PV	PCV %	EV	ECV %	h^2bs	GA%
**Ch.c**	66.645*	47.426	13.69	53.727	39.265	90.99	25.575	73.434	34.86	8.085
**CD**	0.340*	1.316	0.092	26.485	0.155	34.327	0.063	21.838	59.52	31.257
**CL**	1.084*	7.276	0.08	10.488	0.924	35.643	0.844	34.065	8.658	2.008
**CW**	854.03*	109.04	18.005	40.635	818.02	273.898	800.015	270.867	2.201	1.013
**CPP**	0.391*	2.861	0.107	19.299	0.178	24.961	0.072	15.83	59.78	15.482
**FLSWR**	1.034*	3.525	0.314	29.822	0.407	33.988	0.094	16.303	76.99	24.459
**NGRC**	2.788*	15	0.404	16.412	1.98	36.329	1.576	32.411	20.41	3.36
**NGPC**	6268.25*	279.25	1863.737	258.342	2540.777	301.638	677.04	155.708	73.35	23.237
**GY**	431433.0*	912.28	143714.33	1255.122	144004.33	1256.388	290	56.381	99.79	72.855
**LA**	15892.40*	710.54	3265.167	214.367	9362.067	362.987	6096.9	292.928	34.88	8.335
**Nlp**	4.614*	11.583	1.51	36.107	1.593	37.09	0.083	8.48	94.77	18.126
**OC**	0.761*	4.154	0.142	18.464	0.477	33.899	0.336	28.429	29.67	8.66
**PH**	762.379*	239.77	241.74	100.41	278.898	107.851	37.158	39.367	86.68	10.595
**PC**	1.090*	7.814	0.319	20.217	0.452	24.042	0.132	13.012	70.71	10.673
**SD**	0.020*	0.609	0.006	10.271	0.007	10.508	0	2.219	95.54	22.574
**TDM**	39631.90*	1195.1	6430.3	231.96	26771.3	473.296	20341	412.557	24.02	5.771

Table S1d. Correlation among grain yield and its attributing traits during crop growing season 1 (February 2010-2011) in maize hybrids under water stress environment.

Traits	Chlorophyll contents	Cob diameter	Cob length	Cob weight	Cobs per plant	Fresh leaf/stem weight ratio	Grain rows/cob	Grains per cob	Grain yield/plant	Leaf area	Leaves per plant	Oil contents	Plant height	Protein contents	Stem diameter
**Cob diameter**	0.66*														
**P<0.05**	0.00														
**Cob length**	0.72*	0.72													
**P<0.05**	0.00	0.00													
**Cob weight**	0.3688*	0.1974	-0.0871												
**P<0.05**	0.0902	0.2485	0.6136												
**Cobs per plant**	0.782	-0.0096	-0.257	0.6033											
**P<0.05**	0.6502*	0.9556	0.1302	0.0001											
**Fresh leaf/stem weight ratio**	0.5931*	0.7735	0.503	0.3211	0.0653										
**P<0.05**	0.0001	0	0.0018	0.0562	0.705										
**Grain rows/cob**	0.639*	0.6093	0.5703	-0.04	-0.0269	0.5434									
**P<0.05**	0	0.0001	0.0003	0.817	0.8762	0.0006									
**Grains per cob**	0.4863*	0.6197	0.2976	0.298	0.0712	0.6997	0.49								
**P<0.05**	0.0026	0.0001	0.078	0.0775	0.68	0	0.0024								
**Grain yield/plant**	0.9146*	0.8103*	0.8606*	0.954*	0.282*	0.6766*	0.5822*	0.666*							
**P<0.05**	0.0119	0.0001	0.0307	0.0341	0.0956	0	0.0002	0							
**Leaf area**	0.2664*	0.443*	-0.1776	0.2856	0.0704	0.5965	0.0772	0.6398	0.7647*						
**P<0.05**	0.1163	0.0068	0.3001	0.0913	0.6834	0.0001	0.6546	0	0.0043						
**Leaves per plant**	0.3109*	0.4634	-0.2186*	0.3369	0.1502	0.2598	0.3172	0.2115	0.2756*	-0.014					
**P<0.05**	0.0649	0.0044	0.2002	0.0445	0.3818	0.1259	0.0594	0.2155	0.1037	0.9354					
**Oil contents**	0.2967*	0.4416	0.2854*	-0.1459	-0.3155	0.4331	0.185	0.5465	0.3252*	0.4292	0.0992				
**P<0.05**	0.0789	0.007	0.0916	0.3957	0.0609	0.0083	0.28	0.0006	0.053	0.009	0.565				
**Plant height**	0.215*	0.4982	-0.1267	0.423	0.2058	0.5538	0.2166	0.5357	0.5222*	0.4125	0.611	0.392			
**P<0.05**	0.2079	0.002	0.4613	0.0102	0.2284	0.0005	0.2045	0.0008	0.0011	0.0124	0.0001	0.0181			
**Protein contents**	0.4061*	0.4987	-0.2278*	0.3907	0.3207	0.6553	0.3019	0.6047	0.7053*	0.5572	0.137	0.3736	0.4728		
**P<0.05**	0.014	0.002	0.1815	0.0185	0.0565	0	0.0736	0.0001	0	0.0004	0.4257	0.0248	0.0036		
**Stem diameter**	0.4143*	0.6598	0.279	0.3382	0.2854	0.5826	0.4763	0.4751	0.6046*	0.3003	0.6833	0.4402	0.6925	0.492	
**P<0.05**	0.012	0	0.0993	0.0436	0.0915	0.0002	0.0033	0.0034	0.0001	0.0751	0	0.0072	0	0.0023	
**Total dry matter**	0.582*	0.5352	0.6361*	0.074	0.0111	0.4328	0.5237	0.2854	0.4586*	0.3327	0.3305	0.2751	0.3046	0.3168	0.4948
**P<0.05**	0.0002	0.0008	0	0.668	0.9486	0.0084	0.001	0.0916	0.0049	0.0474	0.049	0.1044	0.0709	0.0598	0.0022

*= Significant at 5% probability level

(Suppl. Table S2a). Duncan Multiple Range Test (DMRT) of under studies traits in twelve maize hybrids during crop growing season 2 (August 2010-2011) [see section materials and methods for hybrid codes and traits description].

Traits													
Ch.c	Hybrids	H9	H6	H12	H10	H11	H5	H3	H7	H8	H4	H2	H1
	DMRT Values	61.000	56.767	55.670	55.100	54.533	54.067	53.300	53.200	52.967	52.567	48.833	48.067
	Pair wise comparison	A	AB	AB	ABC	ABCD	BCD	BCD	BCD	BCD	BCD	CD	D
CD	Hybrids	H9	H6	H5	H8	H7	H4	H2	H3	H1	H11	H12	H10
	DMRT Values	2.2633	1.8333	1.8000	1.8000	1.7667	1.6667	1.4633	1.3033	1.2467	1.2467	1.2067	1.1600
	Pair wise comparison	A	AB	AB	AB	AB	AB	B	B	B	B	B	B
CL	Hybrids	H9	H11	H4	H7	H10	H8	H6	H12	H2	H5	H3	H1
	DMRT Values	9.1033	8.1000	8.0667	8.0000	7.9000	7.7000	7.4667	7.2767	7.1667	7.1667	6.9667	6.5300
	Pair wise comparison	A	AB	AB	AB	AB	AB	AB	B	B	B	B	B
CW	Hybrids	H9	H12	H6	H5	H11	H8	H2	H3	H7	H4	H10	H1
	DMRT Values	165.50	131.93	128.97	127.60	125.70	120.60	118.77	118.73	108.10	106.53	101.07	97.60
	Pair wise comparison	A	AB	BC	BC	BC	BC	BC	BC	BC	BC	BC	C
CPP	Hybrids	H9	H12	H1	H10	H3	H5	H11	H2	H4	H6	H7	H8
	DMRT Values	3.6667	3.6667	3.3333	3.3333	3.3333	3.0000	2.6667	2.6667	2.6667	2.6667	2.6667	2.6667
	Pair wise comparison	A	A	A	A	A	A	A	A	A	A	A	A
FLSWR	Hybrids	H9	H11	H12	H4	H10	H8	H3	H5	H7	H6	H1	H2
	DMRT Values	5.3500	4.0033	3.8600	3.5000	3.4467	3.3667	3.2533	3.2067	3.1500	3.0200	2.9900	2.8800
	Pair wise comparison	A	B	BC	BCD	BCD	BCD	BCD	BCD	BCD	CD	CD	D
NGRPC	Hybrids	H9	H8	H6	H7	H10	H4	H3	H5	H11	H1	H12	H2
	DMRT Values	18.667	17.333	16.667	16.000	14.667	14.667	14.000	14.000	13.333	12.667	12.667	12.667
	Pair wise comparison	A	AB	ABC	ABCD	BCDE	BCDE	CDE	CDE	DE	E	E	E
NGPC	Hybrids	H9	H12	H11	H10	H8	H5	H7	H6	H4	H3	H1	H2
	DMRT Values	445.00	370.00	361.67	332.00	313.67	301.67	301.00	294.67	269.67	213.33	211.67	209.67

	Pair wise comparison	A	B	B	BC	C	CD	CD	CD	D	E	E	E
LA	Hybrids	H9	H12	H10	H11	H8	H6	H4	H5	H1	H7	H2	H3
	DMRT Values	848.00	787.47	719.28	682.25	679.58	648.33	627.88	621.67	596.24	584.24	581.33	556.07
	Pair wise comparison	A	AB	BC	BCD	BCD	CDE	CDE	CDE	DE	DE	DE	E
nlp	Hybrids	H9	H12	H1	H6	H7	H8	H11	H5	H10	H3	H4	H2
	DMRT Values	15.000	14.333	12.000	12.000	12.000	12.000	11.000	11.000	10.000	10.000	10.000	9.000
	Pair wise comparison	A	B	C	C	C	C	D	D	E	E	E	F
OC	Hybrids	H9	H11	H10	H12	H4	H8	H7	H6	H2	H5	H1	H3
	DMRT Values	5.6667	5.0667	4.8200	4.7700	4.1333	4.1267	4.1000	3.7000	3.6433	3.6400	3.4633	3.3667
	Pair wise comparison	A	AB	B	BC	CD	CD	CD	DE	DE	DE	DE	E
PH	Hybrids	H9	H12	H8	H5	H7	H1	H6	H3	H4	H10	H11	H2
	DMRT Values	281.00	272.50	249.10	241.20	238.10	232.10	221.30	221.20	216.50	214.20	209.50	201.10
	Pair wise comparison	A	B	C	D	E	F	G	G	H	I	J	K
PC	Hybrids	H9	H12	H10	H5	H7	H6	H11	H8	H3	H2	H1	H4
	DMRT Values	9.5467	8.2833	7.8733	7.8333	7.7733	7.7600	7.7333	7.5567	7.0000	6.7233	6.6667	6.1333
	Pair wise comparison	A	B	BC	BC	BC	BC	BC	BC	CD	CD	CD	D
SD	Hybrids	H9	H3	H12	H1	H7	H2	H5	H8	H6	H10	H4	H11
	DMRT Values	0.8500	0.7100	0.6400	0.6200	0.6000	0.5600	0.5600	0.5467	0.5300	0.5000	0.4900	0.4400
	Pair wise comparison	A	B	C	D	E	F	F	FG	G	H	H	I
TDM	Hybrids	H9	H4	H10	H12	H8	H3	H5	H7	H11	H6	H1	H2
	DMRT Values	1511.7	1344.7	1341.7	1339.7	1319.0	1277.1	1256.0	1255.8	1237.3	1211.5	1180.0	1081.8
	Pair wise comparison	A	AB	AB	AB	AB	BC	BC	BC	BC	BC	BC	C
GY	Hybrids	H9	H12	H10	H5	H8	H1	H3	H11	H4	H7	H6	H2
	DMRT Values	1715.0	1507.0	1224.0	1212.0	1035.0	856.0	848.0	690.0	608.0	578.0	490.0	398.0
	Pair wise comparison	A	B	C	C	D	E	E	F	G	G	H	I

Supplementary Table S2b: ANOVA (Analysis of variance) of all studied traits in twelve maize hybrids during crop growing season 2 (August 2010-2011) of grain yield and its attributing traits.

Randomized Complete Block AOV Table for Chlorophyll contents

Source	DF	SS	MS	F	P
repli	2	8.529	4.2646		
hybrids	11	380.349	34.5772	2.22	0.0005
Error	22	343.095	15.5952		
Total	35	731.973			

Grand Mean 53.839   CV 7.33

Randomized Complete Block AOV Table for Cob diameter

Source	DF	SS	MS	F	P
repli	2	0.19049	0.09524		
hybrids	11	3.88496	0.35318	2.18	0.0000
Error	22	3.56371	0.16199		
Total	35	7.63916			

Grand Mean 1.5631   CV 25.75

Randomized Complete Block AOV Table for Cob length

Source	DF	SS	MS	F	P
repli	2	0.4203	0.21014		
hybrids	11	15.0802	1.37093	1.31	0.0004
Error	22	22.9386	1.04266		
Total	35	38.4391			

Grand Mean 7.6203   CV 13.40

Randomized Complete Block AOV Table for Cob weight

Source	DF	SS	MS	F	P
repli	2	400.5	200.241		
hybrids	11	10679.1	970.832	2.39	0.0003
Error	22	8945.3	406.605		
Total	35	20024.9			

Grand Mean 120.92   CV 16.68

Randomized Complete Block AOV Table for cobs per plant

Source	DF	SS	MS	F	P
repli	2	0.8889	0.44444		
hybrids	11	20.4444	1.85858	7.2515	0.0003
Error	22	5.6389	0.25631		
Total	35	26.9722			

Grand Mean 3.0278   CV 31.84

Randomized Complete Block AOV Table for fresh leaf to stem weight ratio

Source	DF	SS	MS	F	P
repli	2	4.0137	2.00687		
hybrids	11	14.9109	1.35554	4.75	0.0009
Error	22	6.2828	0.28558		
Total	35	25.2074			

Grand Mean 3.5022   CV 15.26

Randomized Complete Block AOV Table for grain rows per cob

Source	DF	SS	MS	F	P
repli	2	2.889	1.4444		
hybrids	11	130.222	11.8384	4.26	0.0019
Error	22	61.111	2.7778		
Total	35	194.222			

Grand Mean 14.778   CV 11.28

Randomized Complete Block AOV Table for grains per cob

Source	DF	SS	MS	F	P
repli	2	3096	1548.1		
hybrids	11	165951	15086.4	24.41	0.0000
Error	22	13599	618.1		
Total	35	182646			

Grand Mean 302.00   CV 8.23

Randomized Complete Block AOV Table for grain yield per plant

Source	DF	SS	MS	F	P
repli	2	3095	1548		
hybrids	11	5700453	518223	271.69	0.0000
Error	22	41963	1907		
Total	35	5745511			

Grand Mean 930.08   CV 4.70

Randomized Complete Block AOV Table for leaf area

Source	DF	SS	MS	F	P
repli	2	53639	26819.6		
hybrids	11	256213	23292.1	4.95	0.0007
Error	22	103465	4703.0		
Total	35	413318			

Grand Mean 661.03   CV 10.37

Randomized Complete Block AOV Table for leaf per plant

Source	DF	SS	MS	F	P
repli	2	0.056	0.02778		
hybrids	11	104.306	9.48232	341.36	0.0000
Error	22	0.611	0.02778		
Total	35	104.972			

Grand Mean 11.528   CV 1.45

Randomized Complete Block AOV Table for oil contents

Source	DF	SS	MS	F	P
repli	2	0.1537	0.07685		
hybrids	11	17.2235	1.56577	9.64	0.0000
Error	22	3.5732	0.16242		
Total	35	20.9504			

Grand Mean 4.2081   CV 9.58

Randomized Complete Block AOV Table for plant height

Source	DF	SS	MS	F	P
repli	2	0.2	0.08		
hybrids	11	20066.8	1824.25	21891.0	0.0000
Error	22	1.8	0.08		
Total	35	20068.8			

Grand Mean 233.15   CV 0.12

Randomized Complete Block AOV Table for protein contents

Source	DF	SS	MS	F	P
repli	2	0.0086	0.00431		
hybrids	11	25.8100	2.34636	4.24	0.0019
Error	22	12.1658	0.55299		
Total	35	37.9844			

Grand Mean 7.5736   CV 9.82

Randomized Complete Block AOV Table for stem **diameter**

Source	DF	SS	MS	F	P
repli	2	0.00044	0.00022		
hybrids	11	0.39986	0.03635	282.81	0.0000
Error	22	0.00283	0.00013		
Total	35	0.40312			

Grand Mean 0.5872   CV 1.93

Randomized Complete Block AOV Table for total dry matter

Source	DF	SS	MS	F	P
repli	2	15289	7644.7		
hybrids	11	371152	33741.1	1.92	0.0936
Error	22	387478	17612.6		
Total	35	773920			

Grand Mean 1279.7   CV 10.37

Table S2c. Genetic Components for under studied traits of twelve maize hybrids during crop growing season 2 (August 2010-2011) under stress environment.

Traits	M.S	G.M	GV	GCV %	PV	PCV %	EV	ECV %	h^2bs%	GA%
**Ch.c**	34.577*	53.839	6.327	34.282	21.923	63.811	15.595	53.820	50.862	7.405
**CD**	0.353*	1.563	0.064	20.198	0.226	37.996	0.162	32.183	64.257	28.071
**CL**	1.371*	7.620	0.109	11.984	1.152	38.882	1.043	36.989	9.499	8.348
**CW**	970.832*	120.920	188.076	124.715	594.681	221.765	406.605	183.374	13.626	11.194
**CPP**	0.513*	3.028	0.140	21.495	0.233	27.729	0.093	17.516	60.095	16.806
**FLSWR**	1.356*	3.502	0.357	31.911	0.642	42.822	0.286	28.556	55.532	22.301
**NGRC**	11.838*	14.778	3.020	45.207	5.798	62.637	2.778	43.355	52.090	16.896
**NGPC**	15086.4*	302.000	4822.767	399.618	5440.867	424.454	618.100	143.063	88.640	37.996
**GY**	518223.0*	930.080	172105.333	1360.307	174012.33	1367.823	1907.00	143.191	98.904	77.850
**LA**	23292.1*	661.030	6196.367	306.167	10899.367	406.060	4703.00	266.733	56.851	15.758
**Nlp**	9.482*	11.528	3.152	52.286	3.179	52.516	0.028	4.909	99.126	19.908
**OC**	1.566*	4.208	0.468	33.341	0.630	38.699	0.162	19.646	74.227	24.575
**PH**	1824.25*	233.150	608.057	161.493	608.137	161.504	0.080	1.852	99.987	18.560
**PC**	2.346*	7.574	0.598	28.095	1.151	38.980	0.553	27.021	51.947	12.913
**SD**	0.036*	0.587	0.012	14.339	0.012	14.416	0.0001	1.488	98.935	32.665
**TDM**	33741.1*	1279.700	5376.167	204.966	22988.767	423.842	17612.60	370.986	23.386	9.863

Supplementary Table S2d. Correlation among grain yield and its attributing traits during crop growing season 2 (August 2010-2011) in maize hybrids under stress environment.

**Traits**	**Chlorophyll contents**	**Cob diameter**	**Cob length**	**Cob weight**	**Cobs per plant**	**Fresh leaf/stem weight ratio**	**Grain rows/cob**	**Grains per cob**	**Grain yield/plant**	**Leaf area**	**Leaves per plant**	**Oil contents**	**Plant height**	**Protein contents**	**Stem diameter**
**Cob diameter**	0.38*														
**P<0.05**	0.02														
**Cob length**	0.60*	0.54*													
**P<0.05**	0.00	0.00													
**Cob weight**	0.34*	0.34*	-0.11												
**P<0.05**	0.0458	0.042	0.5153												
**Cobs per plant**	0.4367*	-0.0155	0.3472*	-0.1522											
**P<0.05**	0.0077	0.9283	0.038	0.3757											
**Fresh leaf/stem weight ratio**	0.4602*	0.1315	0.4986*	0.2626*	0.3424*										
**P<0.05**	0.0047	0.4446	0.002	0.1218	0.0409										
**Grain rows/cob**	0.344*	0.5341*	0.4913*	0.0748	-0.0384	0.2589*									
**P<0.05**	0.0399	0.0008	0.0023	0.6646	0.8241	0.1273									
**Grains per cob**	0.5548*	0.1957	0.4751*	0.4256*	0.2145*	0.7517*	0.3378*								
**P<0.05**	0.0004	0.2528	0.0034	0.0096	0.2091	0	0.0439								
**Grain yield/plant**	0.8901*	0.8384*	0.7915*	0.8047*	0.3649*	0.5243*	0.9133*	0.6485*							
**P<0.05**	0.0107	0.0207	0.2385	0.0143	0.0287	0.001	0.0393	0							
**Leaf area**	0.3528*	0.0345	0.1593	0.4914*	0.2122	0.6998*	0.0926	0.7583*	0.9186*						
**P<0.05**	0.0348	0.8418	0.3533	0.0023	0.2142	0	0.591	0	0.0001						
**Leaves per plant**	0.412*	-0.213	-0.1709	0.4237*	0.2532*	0.4376*	0.2187*	0.6433*	0.6523*	0.5626*					
**P<0.05**	0.0125	0.2123	0.319	0.01	0.1362	0.0076	0.2001	0	0	0.0004					
**Oil contents**	0.3872*	0.0243	0.5394*	0.2551*	0.1128	0.7157*	-0.2147*	0.8212*	0.5544*	0.6119*	0.4507*				
**P<0.05**	0.0196	0.888	0.0007	0.1333	0.5124	0	0.2086	0	0.0005	0.0001	0.0058				
**Plant height**	0.4056*	0.3298*	-0.1989	0.4606*	0.2783*	0.4846*	-0.2977*	0.6166*	0.8061*	0.5428*	0.9016*	0.4158*			
**P<0.05**	0.0141	0.0495	0.2449	0.0047	0.1003	0.0027	0.0778	0.0001	0	0.0006	0	0.0117			
**Protein contents**	0.5537*	-0.1678	0.1158	0.5678*	0.2573*	0.4145*	-0.1029	0.6517*	0.8598*	0.7293*	0.5836*	0.4748*	0.5929*		
**P<0.05**	0.0005	0.3279	0.5012	0.0003	0.1298	0.012	0.5503	0	0.0001	0.0009	0.0002	0.0034	0.0001		
**Stem diameter**	0.2692*	-0.3038*	-0.1178	0.4408*	0.3193*	0.4283*	0.1444	0.2433*	0.5685*	-0.2936*	0.5262*	0.1739	0.6892*	0.4171*	
**P<0.05**	0.1124	0.0716	0.4938	0.0071	0.0577	0.0092	0.4008	0.1528	0.0003	0.0822	0.001	0.3103	0	0.0114	
**Total dry matter**	0.5846*	0.3475*	0.6885*	0.0552	0.5687*	0.6394*	0.3631*	0.5904*	0.5209*	0.46*	0.3803*	0.4658*	0.4762*	0.1898	0.3487*
**P<0.05**	0.0002	0.0378	0	0.7493	0.0003	0	0.0295	0.0002	0.0011	0.0048	0.0221	0.0042	0.0033	0.2676	0.0372

*= Significant at 5% probability level

Supplementary Table S3a: Duncan Multiple Range Test (DMRT) of under studies traits in twelve maize hybrids during crop growing season 3 (February 2011-2012) [see section materials and methods for hybrid codes and traits description].

Traits													
Ch.c	Hybrids	H9	H11	H10	H8	H3	H7	H6	H4	H12	H5	H2	H1
	DMRT Values	61.067	57.433	55.400	55.233	53.867	53.700	53.233	52.867	52.700	51.900	49.000	47.000
	Pair wise comparison	A	AB	ABC	ABC	ABCD	ABCD	BCD	BCD	BCD	BCD	CD	D
CD	Hybrids	H9	H2	H3	H4	H8	H10	H11	H7	H6	H12	H5	H1
	DMRT Values	2.4333	1.9667	1.8967	1.8000	1.8000	1.7333	1.7000	1.6667	1.6333	1.5200	1.3967	1.2800
	Pair wise comparison	A	AB	ABC	ABC	ABC	BC	BC	BC	BC	BC	BC	C
CL	Hybrids	H9	H8	H2	H3	H6	H7	H10	H4	H5	H11	H12	H1
	DMRT Values	9.2133	8.4333	8.2667	8.2667	8.2333	8.1000	8.0000	7.8667	7.8667	7.4333	7.4333	6.8100
	Pair wise comparison	A	AB	ABC	ABC	ABC	ABC	ABC	ABC	ABC	BC	BC	C
CW	Hybrids	H9	H3	H10	H4	H5	H11	H7	H8	H2	H12	H6	H1
	DMRT Values	166.33	150.33	149.70	145.63	144.83	144.13	142.17	139.53	138.30	133.60	131.70	100.17
	Pair wise comparison	A	AB	AB	AB	AB	AB	AB	AB	AB	ABC	BC	C
CPP	Hybrids	H9	H10	H2	H3	H7	H1	H11	H6	H8	H12	H4	H5
	DMRT Values	3.6667	3.3333	3.3333	3.3333	3.3333	3.0000	3.0000	3.0000	3.0000	2.6667	2.6667	2.6667
	Pair wise comparison	A	A	A	A	A	A	A	A	A	A	A	A
FLSWR	Hybrids	H9	H12	H11	H10	H5	H6	H8	H7	H1	H4	H2	H3
	DMRT Values	5.0767	4.4533	3.8400	3.5700	3.3800	3.3367	3.1300	3.0533	3.0333	2.9367	2.8167	2.6033
	Pair wise	A	AB	BC	CD	CDE	CDE	CDEF	DEF	DEF	DEF	EF	F

	comparison												
NGRPC	Hybrids	H9	H12	H7	H4	H6	H8	H5	H11	H2	H3	H1	H10
	DMRT Values	19.333	16.667	16.667	16.000	16.000	16.000	15.333	14.667	14.667	14.667	14.000	14.000
	Pair wise comparison	A	B	B	BC	BC	BC	BC	BC	BC	BC	C	C
NGPC	Hybrids	H9	H12	H11	H10	H3	H4	H8	H5	H7	H6	H2	H1
	DMRT Values	472.33	377.67	375.33	349.33	347.00	335.33	332.67	317.67	303.00	302.00	298.00	203.67
	Pair wise comparison	A	B	B	BC	BC	BC	BC	BC	C	C	C	D
LA	Hybrids	H9	H12	H11	H8	H7	H10	H5	H6	H4	H1	H3	H2
	DMRT Values	851.33	787.13	736.65	720.81	699.63	698.60	692.59	677.75	651.75	586.92	580.79	553.83
	Pair wise comparison	A	AB	BC	BCD	CD	CD	CD	CD	DE	EF	EF	F
nlp	Hybrids	H9	H4	H3	H5	H6	H7	H1	H10	H12	H2	H11	H8
	DMRT Values	14.333	13.000	12.000	12.000	12.000	12.000	11.000	11.000	11.000	11.000	10.000	10.000
	Pair wise comparison	A	B	C	C	C	C	D	D	D	D	E	E
OC	Hybrids	H9	H12	H6	H7	H11	H10	H5	H8	H4	H3	H1	H2
	DMRT Values	5.7833	5.6200	5.1767	5.0967	5.0567	4.9333	4.8367	4.8333	4.2733	3.8900	3.7667	3.6300
	Pair wise comparison	A	AB	ABC	ABC	ABC	BCD	CD	CD	DE	E	E	E
PH	Hybrids	H9	H4	H5	H10	H12	H7	H3	H2	H11	H6	H1	H8
	DMRT Values	282.40	279.33	275.60	260.50	253.50	244.60	241.20	221.20	208.90	205.90	196.40	177.50
	Pair wise comparison	A	B	C	D	E	F	G	H	I	J	K	L
PC	Hybrids	H9	H7	H8	H6	H5	H10	H4	H11	H3	H12	H2	H1
	DMRT	8.6333	8.5000	8.2200	8.1333	8.1000	8.0667	8.0667	7.9200	7.7667	7.2267	7.0167	6.7533

	Values												
	Pair wise comparison	A	AB	ABC	ABC	ABCD	ABCD	ABCD	ABCD	ABCD	BCD	CD	D
SD	Hybrids	H9	H5	H11	H12	H7	H8	H10	H4	H6	H3	H1	H2
	DMRT Values	0.8267	0.7100	0.6900	0.5800	0.5800	0.5467	0.5200	0.5200	0.5100	0.4900	0.4400	0.4300
	Pair wise comparison	A	B	B	C	C	D	E	E	EF	F	G	G
TDM	Hybrids	H9	H10	H8	H5	H4	H6	H7	H3	H11	H12	H1	H2
	DMRT Values	1607.3	1437.7	1388.7	1380.3	1370.3	1348.7	1322.0	1314.3	1307.3	1269.7	1267.0	1245.3
	Pair wise comparison	A	B	BC	BC	BC	BCD	CDE	CDE	CDE	DE	DE	E
GY	Hybrids	H9	H3	H12	H10	H2	H11	H7	H8	H4	H6	H5	H1
	DMRT Values	1740.3	1604.0	1557.7	1472.0	1380.0	1134.0	1112.0	1068.0	904.0	867.0	712.0	624.0
	Pair wise comparison	A	B	B	C	D	E	E	E	F	F	G	H

Supplementary Table S3b: ANOVA (Analysis of variance) of all studied traits in twelve maize hybrids during crop growing season 3 (February 2011-2012) of grain yield and its attributing traits.

Randomized Complete Block AOV Table for Chlorophyll contents

Source	DF	SS	MS	F	P
repli	2	33.095	16.5475		
hybrids	11	436.570	39.6882	1.93	0.0001
Error	22	452.265	20.5575		
Total	35	921.930			

Grand Mean 53.617   CV 8.46

Randomized Complete Block AOV Table for Cob diameter

Source	DF	SS	MS	F	P
repli	2	0.01087	0.00544		
hybrids	11	2.87962	0.26178	1.73	0.0001
Error	22	3.32279	0.15104		
Total	35	6.21329			

Grand Mean 1.7356   CV 22.39

Randomized Complete Block AOV Table for Cob length

Source	DF	SS	MS	F	P
repli	2	0.1219	0.06094		
hybrids	11	11.8800	1.08000	1.34	0.0002
Error	22	17.7511	0.80687		
Total	35	29.7530			

Grand Mean 7.9936   CV 11.24

Randomized Complete Block AOV Table for Cob weight

Source	DF	SS	MS	F	P
repli	2	44.0	22.005		
hybrids	11	8002.2	727.470	1.85	0.0010
Error	22	8627.8	392.174		
Total	35	16674.0			

Grand Mean 140.54   CV 14.09

Randomized Complete Block AOV Table for cobs per plant

Source	DF	SS	MS	F	P
repli	2	0.1667	0.08333		
hybrids	11	19.1667	1.7424	11.2195	0.0001
Error	22	3.4167	0.1553		
Total	35	22.7500			

Grand Mean 3.0833 CV 30.27

Randomized Complete Block AOV Table for fresh leaf to stem weight ratio

Source	DF	SS	MS	F	P
repli	2	5.9563	2.97813		
hybrids	11	16.9481	1.54074	8.73	0.0000
Error	22	3.8849	0.17658		
Total	35	26.7893			

Grand Mean 3.4358 CV 12.23

Randomized Complete Block AOV Table for grain rows per cob

Source	DF	SS	MS	F	P
repli	2	3.294E-29	1.647E-29		
hybrids	11	73.3333	6.66667	3.44	0.0066
Error	22	42.6667	1.93939		
Total	35	116.000			

Grand Mean 15.667 CV 8.89

Randomized Complete Block AOV Table for grains per cob

Source	DF	SS	MS	F	P
repli	2	12835	6417.3		
hybrids	11	131072	11915.6	8.87	0.0000
Error	22	29563	1343.8		
Total	35	173469			

Grand Mean 334.50 CV 10.96

Randomized Complete Block AOV Table for grain yield per plant

Source	DF	SS	MS	F	P
repli	2	5846	2923		
hybrids	11	4449647	404513	169.47	0.0000
Error	22	52511	2387		
Total	35	4508005			

Grand Mean 1181.3   CV 4.14

Randomized Complete Block AOV Table for leaf area

Source	DF	SS	MS	F	P
repli	2	25615	12807.7		
hybrids	11	243972	22179.3	9.00	0.0000
Error	22	54222	2464.6		
Total	35	323809			

Grand Mean 686.48   CV 7.23

Randomized Complete Block AOV Table for leaf per plant

Source	DF	SS	MS	F	P
repli	2	0.0556	0.02778		
hybrids	11	49.8889	4.53535	163.27	0.0000
Error	22	0.6111	0.02778		
Total	35	50.5556			

Grand Mean 11.611   CV 1.44

Randomized Complete Block AOV Table for oil contents

Source	DF	SS	MS	F	P
repli	2	0.6614	0.33068		
hybrids	11	16.3688	1.48808	7.32	0.0000
Error	22	4.4712	0.20324		
Total	35	21.5014			

Grand Mean 4.7414   CV 9.51

Randomized Complete Block AOV Table for plant height

Source	DF	SS	MS	F	P
repli	2	1.1	0.53		
hybrids	11	40312.3	3664.75	6943.75	0.0000
Error	22	11.6	0.53		
Total	35	40325.0			

Grand Mean 237.25   CV 0.31

Randomized Complete Block AOV Table for protein contents

Source	DF	SS	MS	F	P
repli	2	0.2044	0.10218		
hybrids	11	11.1112	1.01011	1.58	0.1732
Error	22	14.0494	0.63861		

Total	35	25.3650

Grand Mean 7.8669   CV 10.16

Randomized Complete Block AOV Table for stem diameter

Source	DF	SS	MS	F	P
repli	2	0.00061	0.00030		
hybrids	11	0.45636	0.04149	178.00	0.0000
Error	22	0.00513	0.00023		
Total	35	0.46210			

Grand Mean 0.5703   CV 2.68

Randomized Complete Block AOV Table for total dry matter

Source	DF	SS	MS	F	P
repli	2	1784	891.9		
hybrids	11	313871	28533.7	8.36	0.0000
Error	22	75069	3412.2		
Total	35	390724			

Grand Mean 1354.9   CV 4.31

Supplementary Table S3c. Genetic Components for under studied traits of twelve maize hybrids during crop growing season 3 (February 2011-2012) under stress environment.

Traits	M.S	G.M	GV	GCV %	PV	PCV %	EV	ECV %	h^2bs%	GA%
**Ch.c**	39.688*	53.617	6.377	34.487	26.934	70.877	20.558	61.920	48.676	7.022
**CD**	0.262*	1.736	0.037	14.585	0.188	32.902	0.151	29.493	61.650	29.611
**CL**	1.080*	7.994	0.091	10.669	0.898	33.516	0.807	31.773	10.134	9.108
**CW**	727.47*	140.540	111.765	89.177	503.939	189.360	392.174	167.047	11.178	12.217
**CPP**	0.311*	3.083	0.075	15.562	0.162	22.899	0.087	16.799	46.186	13.571
**FLSWR**	1.541*	3.436	0.455	36.376	0.632	42.876	0.177	22.697	71.979	29.220
**NGRC**	6.667*	15.667	1.576	31.716	3.515	47.366	1.939	35.180	44.836	15.416
**NGPC**	11915.6*	334.500	3523.933	324.576	4867.733	381.474	1343.800	200.433	72.394	30.500
**GY**	404513.0*	1181.300	134042.000	1065.223	136429.000	1074.665	2387.000	142.150	98.250	79.914
**LA**	22179.3*	686.480	6571.567	309.400	9036.167	362.809	2464.600	189.478	72.725	17.674
**Nlp**	4.535*	11.611	1.502	35.971	1.530	36.304	0.028	4.911	98.170	18.356
**OC**	1.488*	4.741	0.428	30.058	0.631	36.492	0.203	20.693	67.846	19.955
**PH**	3664.75*	237.250	1221.407	226.896	1221.937	226.945	0.530	4.726	99.957	20.847
**PC**	1.010*	7.867	0.124	12.538	0.763	31.136	0.639	28.500	16.215	13.159
**SD**	0.042*	0.570	0.014	15.635	0.014	15.747	0.000	1.873	98.585	36.086
**TDM**	28533.7*	1354.900	8373.833	248.604	11786.033	294.938	3412.200	158.695	71.049	9.991

Supplementary Table S3d. Correlation among grain yield and its attributing traits during crop growing season 3 (February 2011-2012) in maize hybrids under stress environment.

Traits	Chlorophyll contents	Cob diameter	Cob length	Cob weight	Cobs per plant	Fresh leaf/stem weight ratio	Grain rows/cob	Grains per cob	Grain yield/plant	Leaf area	Leaves per plant	Oil contents	Plant height	Protein contents	Stem diameter
**Cob diameter**	0.6381*														
**P<0.05**	0.00														
**Cob length**	0.5525*	0.83*													
**P<0.05**	0.0005	0.00													
**Cob weight**	0.5849*	0.68*	0.68*												
**P<0.05**	0.0002	0.00	0.00												
**Cobs per plant**	0.4347*	0.6462*	0.706*	0.5504*											
**P<0.05**	0.0081	0	0	0.0005											
**Fresh leaf/stem weight ratio**	0.2856*	-0.1817	-0.1714	0.2672*	0.0617										
**P<0.05**	0.0913	0.2889	0.3175	0.1152	0.7209										
**Grain rows/cob**	0.4978*	0.48*	0.4515*	0.3234*	0.292*	0.4006*									
**P<0.05**	0.002	0.003	0.0057	0.0543	0.084	0.0155									
**Grains per cob**	0.462*	0.4358*	0.3082*	0.5731*	0.0315	0.3658*	0.3553*								
**P<0.05**	0.0046	0.0079	0.0675	0.0003	0.8555	0.0283	0.0334								
**Grain yield/plant**	0.7734*	0.8701*	0.9601*	0.7441*	0.2532*	0.3742*	0.2882*	0.624*							
**P<0.05**	0.0249	0.0038	0.031	0.0071	0.1362	0.0245	0.0883	0							
**Leaf area**	0.4454*	0.1863	0.2339*	0.334*	-0.0706	0.6103*	0.4819*	0.5631*	0.8136*						
**P<0.05**	0.0065	0.2767	0.1697	0.0465	0.6826	0.0001	0.0029	0.0004	0.0625						
**Leaves per plant**	0.2341*	0.3525*	0.3419*	0.3203*	-0.1229	0.2457*	0.5397*	0.3508*	0.2976*	0.2293*					
**P<0.05**	0.1694	0.035	0.0412	0.0568	0.4753	0.1486	0.0007	0.0359	0.0243	0.1785					
**Oil contents**	0.3666*	0.1011	0.2117	0.1534	-0.0662	0.575*	0.5177*	0.4881*	0.2569*	0.8289*	0.1968				
**P<0.05**	0.0279	0.5574	0.2152	0.3718	0.7013	0.0002	0.0012	0.0025	0.1303	0	0.2499				
**Plant height**	0.1957*	0.1871	0.1637	0.4094*	-0.0034	0.2718*	0.3049*	0.4277*	-0.3139*	0.2686*	0.7099*	-0.2382*			
**P<0.05**	0.2526	0.2745	0.3402	0.0132	0.9844	0.1088	0.0706	0.0093	0.0623	0.1132	0	0.1618			
**Protein contents**	0.5377*	0.5229*	0.4882*	0.5611*	0.2997*	-0.0535	0.4654*	0.2976*	0.7675*	0.2894*	0.2878*	0.3536*	0.2271*		
**P<0.05**	0.0007	0.0011	0.0025	0.0004	0.0758	0.7569	0.0042	0.0779	0.0959	0.0869	0.0887	0.0344	0.1829		
**Stem diameter**	0.4956*	0.2387*	0.2353*	0.4247*	-0.0522	0.6092*	0.5249*	0.6472*	0.2209*	0.6998*	0.4063*	0.6342*	0.4402*	0.4041*	
**P<0.05**	0.0021	0.1609	0.1671	0.0098	0.7625	0.0001	0.001	0	0.1955	0	0.0139	0	0.0072	0.0145	
**Total dry matter**	0.4946*	0.404*	0.3966*	0.4616*	0.1058	0.4114*	0.5461*	0.5851*	0.2984*	0.4711*	0.5626*	0.4726*	0.4145*	0.4573*	0.6065*
**P<0.05**	0.0022	0.0145	0.0166	0.0046	0.5393	0.0127	0.0006	0.0002	0.0771	0.0037	0.0004	0.0036	0.012	0.0051	0.0001

*= Significant at 5% probability level

Supplementary Table S4a: Duncan Multiple Range Test (DMRT) of under studies traits in twelve maize hybrids during crop growing season 4 (August 2011-2012) [see section materials and methods for hybrid codes and traits description].

Traits													
Ch.c	Hybrids	H9	H11	H12	H6	H8	H5	H10	H7	H4	H3	H2	H1
	DMRT Values	60.533	53.111	52.943	51.900	51.766	50.900	50.801	50.333	49.798	49.557	46.778	44.333
	Pair wise comparison	A	B	B	BC	BC	BC	BC	BC	BC	BC	CD	D
CD	Hybrids	H9	H3	H8	H7	H2	H6	H4	H1	H5	H10	H11	H12
	DMRT Values	2.5333	1.6233	1.6133	1.6033	1.5967	1.5900	1.5567	1.5500	1.5433	1.4933	1.4567	1.2900
	Pair wise comparison	A	B	B	B	B	BC	BC	BC	BC	BC	BC	C
CL	Hybrids	H9	H11	H8	H2	H1	H7	H10	H4	H12	H6	H5	H3
	DMRT Values	9.5400	7.8367	7.7433	7.6133	7.5800	7.5433	7.5200	7.5000	7.4767	7.4533	7.3767	7.3633
	Pair wise comparison	A	B	B	B	B	B	B	B	B	B	B	B
CW	Hybrids	H9	H10	H1	H11	H4	H2	H6	H8	H12	H5	H3	H7
	DMRT Values	162.03	133.93	132.30	130.00	129.51	127.41	125.19	119.73	118.41	116.48	112.39	106.38
	Pair wise comparison	A	AB	AB	AB	AB	AB	AB	AB	AB	AB	B	B
CPP	Hybrids	H9	H1	H10	H11	H2	H3	H5	H6	H7	H8	H12	H4
	DMRT Values	4.0000	3.0000	3.0000	3.0000	3.0000	3.0000	3.0000	3.0000	3.0000	3.0000	2.6667	2.6667
	Pair wise comparison	A	AB	AB	AB	AB	AB	AB	AB	AB	AB	B	B
FLSWR	Hybrids	H9	H11	H12	H4	H7	H10	H5	H6	H1	H3	H2	H8
	DMRT Values	4.4967	4.3667	4.0533	3.8200	3.8100	3.7700	3.6433	3.5533	3.4700	3.3033	3.1833	3.1100
	Pair wise	A	AB	AB	AB	AB	AB	AB	AB	AB	AB	AB	B

	comparison												
NGRPC	Hybrids	H9	H12	H10	H11	H3	H4	H5	H6	H7	H8	H1	H2
	DMRT Values	17.000	16.667	15.333	15.333	15.333	15.000	15.000	15.000	15.000	15.000	14.333	14.000
	Pair wise comparison	A	A	AB	AB	AB	AB	AB	AB	AB	AB	B	B
NGPC	Hybrids	H9	H12	H11	H10	H8	H5	H4	H7	H6	H3	H2	H1
	DMRT Values	461.67	353.67	353.00	341.67	330.33	305.33	305.00	300.67	299.67	281.67	271.67	202.67
	Pair wise comparison	A	B	B	BC	BCD	CDE	CDE	DE	DE	E	E	F
LA	Hybrids	H9	H12	H11	H10	H8	H7	H3	H6	H4	H5	H1	H2
	DMRT Values	854.00	825.67	749.80	719.34	693.52	682.53	681.96	675.72	663.84	655.31	651.33	627.59
	Pair wise comparison	A	AB	BC	CD	CDE	CDE	CDE	CDE	CDE	DE	DE	E
nlp	Hybrids	H9	H1	H12	H4	H5	H6	H10	H3	H7	H8	H11	H2
	DMRT Values	14.667	12.000	12.000	12.000	12.000	12.000	11.000	11.000	11.000	11.000	10.000	10.000
	Pair wise comparison	A	B	B	B	B	B	C	C	C	C	D	D
OC	Hybrids	H12	H11	H10	H8	H9	H7	H6	H5	H4	H3	H2	H1
	DMRT Values	5.5433	5.5300	5.2400	5.1767	5.1433	5.0733	4.7433	4.6400	4.5167	3.9467	3.8833	3.6567
	Pair wise comparison	A	A	AB	ABC	ABC	ABC	BCD	CD	D	E	E	E
PH	Hybrids	H9	H12	H5	H4	H7	H10	H3	H6	H8	H1	H2	H11
	DMRT Values	279.67	255.97	252.27	245.97	243.90	237.63	236.80	224.07	223.33	219.50	214.10	210.27
	Pair wise comparison	A	B	C	D	E	F	F	G	G	H	I	J
PC	Hybrids	H9	H12	H11	H8	H7	H10	H6	H5	H1	H4	H3	H2
	DMRT	9.2333	8.8467	8.2767	8.0100	8.0000	7.9333	7.8100	7.7867	7.4667	7.1800	7.1300	7.0267

	Values<br>Pair wise comparison	A	AB	BC	CD	CD	CD	CDE	CDE	DEF	EF	F	F
SD	Hybrids	H9	H5	H3	H12	H7	H11	H4	H1	H10	H6	H8	H2
	DMRT Values	0.8433	0.7200	0.6100	0.5900	0.5800	0.5700	0.5700	0.5500	0.5500	0.5500	0.5367	0.5100
	Pair wise comparison	A	B	C	CD	D	DE	DE	EF	EF	EF	F	G
TDM	Hybrids	H9	H12	H4	H6	H3	H11	H5	H1	H8	H10	H7	H2
	DMRT Values	1588.3	1302.2	1294.2	1261.8	1250.1	1248.8	1235.2	1234.9	1226.8	1215.8	1181.1	1150.2
	Pair wise comparison	A	B	B	B	B	B	B	B	B	B	B	B
GY	Hybrids	H9	H12	H10	H3	H8	H11	H5	H2	H4	H6	H7	H1
	DMRT Values	1746.3	1544.7	1187.7	1180.0	991.0	958.7	840.0	768.7	767.0	748.3	726.0	687.3
	Pair wise comparison	A	B	C	C	D	D	E	F	F	F	FG	G

Supplementary Table S4b: ANOVA (Analysis of variance) of all studied traits in twelve maize hybrids during crop growing season 4 (August 2011-2012) of grain yield and its attributing traits.

Randomized Complete Block AOV Table for Chlorophyll contents

Source	DF	SS	MS	F	P
repli	2	6.658	3.3290		
hybrids	11	500.288	45.4807	4.82	0.0008
Error	22	207.481	9.4309		
Total	35	714.427			

Grand Mean 51.063 CV 6.01

Randomized Complete Block AOV Table for Cob diameter

Source	DF	SS	MS	F	P
repli	2	0.00375	0.00188		
hybrids	11	3.00708	0.27337	8.43	0.0000
Error	22	0.71325	0.03242		
Total	35	3.72408			

Grand Mean 1.6208 CV 11.11

Randomized Complete Block AOV Table for Cob length

Source	DF	SS	MS	F	P
repli	2	0.0044	0.00220		
hybrids	11	11.5555	1.05050	5.22	0.0005
Error	22	4.4271	0.20123		
Total	35	15.9870			

Grand Mean 7.7122 CV 5.82

Randomized Complete Block AOV Table for Cob weight

Source	DF	SS	MS	F	P
repli	2	236.7	118.344		
hybrids	11	6567.9	597.079	0.77	0.0009
Error	22	17054.6	775.207		
Total	35	23859.1			

Grand Mean 126.15 CV 22.07

Randomized Complete Block AOV Table for cobs per plant

Source	DF	SS	MS	F	P
repli	2	0.0556	0.02778		
hybrids	11	13.2778	1.2071	7.298	0.0009
Error	22	3.6389	0.1654		
Total	35	16.9722			

Grand Mean 3.0278 CV 25.66

Randomized Complete Block AOV Table for fresh leaf to stem weight ratio

Source	DF	SS	MS	F	P
repli	2	6.6840	3.34202		
hybrids	11	14.6900	1.3354	4.7021	0.0002
Error	22	6.2480	0.284		
Total	35	27.6221			

Grand Mean 3.7150 CV 22.00

Randomized Complete Block AOV Table for grain rows per cob

Source	DF	SS	MS	F	P
repli	2	8.0000	4.00000		
hybrids	11	23.4167	2.12879	1.41	0.0002
Error	22	33.3333	1.51515		
Total	35	64.7500			

Grand Mean 15.250 CV 8.07

Randomized Complete Block AOV Table for grains per cob

Source	DF	SS	MS	F	P
repli	2	302	150.8		
hybrids	11	124732	11339.3	21.40	0.0000
Error	22	11655	529.8		
Total	35	136689			

Grand Mean 317.25 CV 7.26

Randomized Complete Block AOV Table for grain yield per plant

Source	DF	SS	MS	F	P
repli	2	2154	1077		
hybrids	11	3872685	352062	385.59	0.0000
Error	22	20087	913		

Total	35	3894926

Grand Mean 1012.1 CV 2.99

Randomized Complete Block AOV Table for leaf area

Source	DF	SS	MS	F	P
repli	2	60042	30021.2		
hybrids	11	161996	14726.9	5.54	0.0003
Error	22	58520	2660.0		
Total	35	280559			

Grand Mean 706.72 CV 7.30

Randomized Complete Block AOV Table for leaves per plant

Source	DF	SS	MS	F	P
repli	2	0.0556	0.02778		
hybrids	11	50.2222	4.56566	164.36	0.0000
Error	22	0.6111	0.02778		
Total	35	50.8889			

Grand Mean 11.556 CV 1.44

Randomized Complete Block AOV Table for oil contents

Source	DF	SS	MS	F	P
repli	2	2.4724	1.23622		
hybrids	11	13.7306	1.24824	11.99	0.0000
Error	22	2.2896	0.10407		
Total	35	18.4926			

Grand Mean 4.7578 CV 6.78

Randomized Complete Block AOV Table for plant height

Source	DF	SS	MS	F	P
repli	2	2.1	1.03		
hybrids	11	13323.5	1211.23	1178.49	0.0000
Error	22	22.6	1.03		
Total	35	13348.2			

Grand Mean 236.96 CV 0.43

Randomized Complete Block AOV Table for protein contents

Source	DF	SS	MS	F	P
repli	2	0.4047	0.20236		
hybrids	11	14.7628	1.34208	8.98	0.0000
Error	22	3.2894	0.14952		
Total	35	18.4569			

Grand Mean 7.8917   CV 4.90

Randomized Complete Block AOV Table for stem diameter

Source	DF	SS	MS	F	P
repli	2	0.00065	0.00032		
hybrids	11	0.28677	0.02607	127.93	0.0000
Error	22	0.00448	0.00020		
Total	35	0.29190			

Grand Mean 0.5983   CV 2.39

Randomized Complete Block AOV Table for total dry matter

Source	DF	SS	MS	F	P
repli	2	9240	4620.1		
hybrids	11	399451	36313.7	1.50	0.0003
Error	22	531835	24174.3		
Total	35	940526			

Grand Mean 1265.8   CV 12.28

Supplementary Table S4c. Genetic Components for under studied traits of twelve maize hybrids during crop growing season 4 (August 2011-2012) under stress environment.

Traits	M.S	G.M	GV	GCV %	PV	PCV %	EV	ECV %	h^2bs%	GA%
**Ch.c**	45.481*	51.063	12.017	48.511	21.448	64.809	9.431	42.976	56.028	8.918
**CD**	0.273*	1.621	0.080	22.262	0.112	26.325	0.032	14.050	71.513	25.950
**CL**	1.051*	7.712	0.283	19.167	0.484	25.060	0.201	16.144	11.500	9.265
**CW**	775.207*	126.150	59.376	68.606	656.455	228.118	597.079	217.557	12.045	12.224
**CPP**	0.604*	3.028	0.091	17.320	0.422	37.324	0.331	33.063	62.533	15.106
**FLSWR**	0.668*	3.715	0.033	9.472	0.601	40.233	0.568	39.102	75.43	28.031
**NGRC**	2.129*	15.250	0.205	11.585	1.720	33.581	1.515	31.519	54.902	16.796
**NGPC**	11339.30*	317.250	3603.167	337.009	4132.967	360.936	529.800	129.228	87.181	33.005
**GY**	352062.0*	1012.100	117049.667	1075.408	117962.667	1079.594	913.000	94.978	99.226	72.095
**LA**	14726.90*	706.720	4022.300	238.569	6682.300	307.496	2660.000	194.007	60.193	16.219
**Nlp**	4.566*	11.556	1.513	36.181	1.541	36.512	0.028	4.905	98.195	18.510
**OC**	2.289*	4.758	0.347	27.006	1.595	57.899	1.248	51.215	62.755	21.134
**PH**	1211.230*	236.960	403.400	130.476	404.430	130.642	1.030	6.593	99.745	19.856
**PC**	1.342*	7.892	0.398	22.447	0.547	26.319	0.149	13.740	72.744	11.960
**SD**	0.026*	0.598	0.009	11.992	0.009	12.131	0.000	1.829	97.727	21.905
**TDM**	36313.70*	1265.800	4046.467	178.795	28220.767	472.174	24174.300	437.013	74.339	9.340

Supplementary Table S4d. Correlation among grain yield and its attributing traits during crop growing season 4 (August 2011-2012) in maize hybrids under stress environment.

Traits	Chlorophyll contents	Cob diameter	Cob length	Cob weight	Cobs per plant	Fresh leaf/stem weight ratio	Grain rows/cob	Grains per cob	Grain yield/plant	Leaf area	Leaves per plant	Oil contents	Plant height	Protein contents	Stem diameter
**Cob diameter**	0.5837*														
**P<0.05**	0.0002														
**Cob length**	0.375*	0.7172*													
**P<0.05**	0.0242	0													
**Cob weight**	0.8368*	0.4103*	0.6426*												
**P<0.05**	0.0312	0.0129	0												
**Cobs per plant**	0.2215*	0.4225*	0.6349*	0.6104*											
**P<0.05**	0.0008	0.0103	0	0.0001											
**Fresh leaf/stem weight ratio**	0.4333*	0.0445	0.1552	0.0179	-0.1014										
**P<0.05**	0.0083	0.7966	0.3662	0.9174	0.5563										
**Grain rows/cob**	0.409*	0.279*	0.4597*	0.2457*	0.3544*	0.3671*									
**P<0.05**	0.0133	0.0994	0.0048	0.1486	0.0339	0.0276									
**Grains per cob**	0.7497*	0.4818*	0.5975*	0.3217*	0.2217*	0.2852*	0.4177*								
**P<0.05**	0	0.0029	0.0001	0.0557	0.1937	0.0917	0.0112								
**Grain yield/plant**	0.9142*	0.8222*	0.9507*	0.8595*	0.26*	0.2467	0.5535*	0.7573*							
**P<0.05**	0.0001	0.0103	0.0005	0.1264	0.1257	0.1469	0.0005	0							
**Leaf area**	0.5029*	-0.234*	0.3542*	-0.0872	-0.0094	0.4642*	0.4511*	0.6809*	0.899*						
**P<0.05**	0.0018	0.1696	0.034	0.6131	0.9565	0.0043	0.0058	0	0						
**Leaves per plant**	0.4872*	0.6402*	0.5822*	0.3266*	0.2873*	0.1398	0.4007*	0.4834*	0.5369*	0.4145*					
**P<0.05**	0.0026	0	0.0002	0.0519	0.0893	0.4162	0.0154	0.0028	0.0007	0.012					
**Oil contents**	0.4504*	-0.0383	0.2359*	-0.0688	-0.0687	0.3701*	0.4297*	0.6549*	0.4192*	0.6677*	0.1139				
**P<0.05**	0.0058	0.8245	0.166	0.6903	0.6904	0.0263	0.0089	0	0.0109	0	0.5083				
**Plant height**	0.5435*	0.5006*	0.4521*	-0.1693	0.2103	-0.1985	0.4751*	0.6062*	0.6725*	0.4637*	0.8021*	0.2727*			
**P<0.05**	0.0006	0.0019	0.0056	0.3236	0.2183	0.2457	0.0034	0.0001	0	0.0044	0	0.1076			
**Protein contents**	0.5192*	0.2292*	0.5161*	-0.0762	0.1705	0.3291*	0.4095*	0.7268*	0.8377*	0.7683*	0.5024*	0.7021*	0.5143*		
**P<0.05**	0.0012	0.1788	0.0013	0.6585	0.32	0.05	0.0131	0	0	0	0.0018	0	0.0013		
**Stem diameter**	0.5868*	0.6774*	0.6205*	0.2771	0.3467*	-0.1847	0.3945*	0.6241*	0.5998*	0.4228*	0.7922*	-0.1887	0.8121*	0.5248*	
**P<0.05**	0.0002	0	0.0001	0.1019	0.0383	0.2809	0.0173	0	0.0001	0.0102	0	0.2704	0	0.001	
**Total dry matter**	0.3212*	0.4544*	0.7713*	0.81*	0.6317*	-0.1481	0.51*	0.5329*	0.4951*	0.3336*	0.5749*	-0.2306	0.4798*	0.3821*	0.5445*
**P<0.05**	0.2146	0.0054	0	0	0	0.3885	0.0015	0.0008	0.0021	0.0468	0.0002	0.176	0.0031	0.0215	0.0006

*= Significant at 5% probability level
